# Closely related species show species-specific environmental responses and different spatial conservation needs: *Prionailurus* cats in the Indian subcontinent

**DOI:** 10.1038/s41598-020-74684-8

**Published:** 2020-10-30

**Authors:** André P. Silva, Shomita Mukherjee, Uma Ramakrishnan, Carlos Fernandes, Mats Björklund

**Affiliations:** 1 Department of Ecology and Genetics, Animal Ecology, Evolutionary Biology Centre, Uppsala University, Norbyvägen 18D, 752 36 Uppsala, Sweden; 2 Sálim Ali Centre for Ornithology and Natural History, Anaikatty Post, Coimbatore, Tamil Nadu 641108 India; 3 National Centre for Biological Sciences, TIFR, Bellary Road, Bangalore, 560065 India; 4 cE3c - Centre for Ecology, Evolution and Environmental Changes, Faculdade de Ciências, Universidade de Lisboa, 1749-016 Lisboa, Portugal; 5 Faculdade de Psicologia, Universidade de Lisboa, Alameda da Universidade, 1649-013 Lisboa, Portugal

**Keywords:** Biogeography, Conservation biology

## Abstract

Phylogenetically closely related species are often assumed to have similar responses to environmental conditions, but species-specific responses have also been described. These two scenarios may have different conservation implications. We tested these two hypotheses for *Prionailurus* cats (*P*. *rubiginosus*, *P*. *bengalensis*, *P*. *viverrinus*) in the Indian subcontinent and show its implications on species current protected area coverage and climatic suitability trends through time. We fitted ecological niche models with current environmental conditions and calculated niche overlap. In addition, we developed a model for the Jungle Cat *Felis chaus* to compare species responses and niche overlap estimates within *Prionailurus* with those for a related sympatric small cat species. Then we estimated the proportion of current suitable environment covered by protected area and projected climatic models from past (last interglacial) to future (2070; RCP4.5 and RCP8.5) conditions to show implications on population management and conservation. The hypothesis of a similar response and niche overlap among closely related species is not supported. Protected area coverage was lowest for *P*. *viverrinus* (mean = 0.071, SD = 0.012) and highest for *P*. *bengalensis* (mean = 0.088, SD = 0.006). In addition, the proportion of the subcontinent with suitable climate varied through time and was species-specific. For *P*. *bengalensis*, climatic suitability shrunk since at least the mid-Holocene, a trend that can be intensified by human-induced climate warming. Concerning *P*. *viverrinus*, most predictions show stable future climatic suitability, but a few indicated potential loss. Climatic suitability for *P*. *rubiginous* was predicted to remain stable but the species exhibited a negative association with intensive agriculture. Similar responses to environmental change by phylogenetically closely related species should not be assumed and have implications on protected area coverage and natural trends of species climatic suitability over time. This should be taken into account during conservation and management actions.

## Introduction

A deep understanding of species responses to the environment is critical to guide spatial conservation strategies under ongoing global change^1,2^. However, paradoxically, such knowledge is often lacking for rare and elusive species as well as for biodiversity rich areas^3–5^. Phylogenetic niche conservatism in its broader sense predicts preservation of ancestral ecological traits among closely related species. Under this assumption, occurrence of common and conspicuous species could potentially be used to predict the distribution and environmental response of closely related species that are more secretive or inhabit remote regions.

The reciprocal geographic distributions of sister taxon pairs of butterflies, birds and mammals in allopatry have been shown to have high congruence^6^. The assumption of phylogenetic niche conservatism has also allowed the discovery of new reptiles based on niche predictions for closely related species^7^. Support for phylogenetic niche conservatism has been found in several other groups including amphibians^8^, freshwater arthropods^9^ and plants^10–12^. However, evidence for species-specific ecological requirements among closely related species has also been widely reported in the literature^13^, and hence assuming conservatism in environmental responses can lead to erroneous conclusions. For example, closely related species of pines^14,15^, tropical amphibians^16,17^, tropical lizards^18–20^ and small mammals^21^ have shown unique niche properties. Evidence is considerably sparser for small endothermic species in the tropics, though not completely absent^22,23^.

Here we evaluate species ecological response and spatial niche conservatism in the presence of environmental change in three small cat species (*Prionailurus* spp.), including two closely related species, in south Asia and implications for protected area coverage. Knowledge about niche conservatism may be informative for conservation planning^24^, for example in the spatial definition of protected areas, in regions with limited baseline biodiversity data, for groups of species whose ecology is little known, or when using more common species as proxies for rarer ones^25–27^. A previous study showed a lack of data for two *Prionalurus* species (*P. planiceps* and *P. viverrinus*)^3^ and an insufficient level of knowledge about species occurrence in the Indian subcontinent^28^, despite this being a priority region as it is extremely biodiverse, including three biodiversity hotspots under various threats^29–31^. This lack of information hinders estimation of species responses to environmental changes and prevents robust planning and evaluation of spatial conservation strategies. Here we go a step further than usual studies on species distribution changes focused on the present time and explore whether a potential lack of niche conservatism can lead to species-specific trends in climatic suitability and their implications for spatial conservation strategies.

To investigate responses of the study species to environmental factors, we developed ecological niche models, including variables related to climate, topography, land cover, human disturbance, and prey occurrence. To test for spatial niche conservatism we estimated niche overlap within the Indian subcontinent for current time, allowing us to include the main environmental facets that can constitute the species niche. We then examined how species ecological affinities (habitat requirements) can influence spatial conservation strategies, namely protected area coverage. Finally, to explore the possibility of different climatic-suitability trends over time, we have also estimated species climatic suitability since the last interglacial (more specifically, for the period 140–120 ka) up to 2070. We used a species of small cat (Jungle Cat, *Felis chaus*) that is sympatric with *Prionailurus* and belongs to a lineage closely related to it (the genus *Felis* is the extant sister clade of the clade containing *Prionailurus*; the two clades diverged at 6.18 Ma^32^) as an outgroup in analyses to test whether environmental response and niche overlap are relatively more similar within *Prionailurus*. Within the Indian subcontinent, climatic niche conservatism has been shown for some species with shared biogeographic histories but not for phylogenetically closely related mammalian species^33^. Although this is the first broad-scale comparative assessment of habitat requirements for all *Prionailurus* species in south Asia, existing expert knowledge on local habitat requirements points to the use of particular environments^34–36^. We therefore expect this pattern to be maintained on a macroscale and visible through species-specific responses.

## Materials and methods

### Study species

We used macroscale comparative analyses to look at potential conservatism of niche properties within the genus *Prionailurus*, which is distributed through Asia and is currently represented by four species, three of them occurring in India (Rusty-spotted Cat *P. rubiginosus,* Leopard Cat *P. bengalensis* and Fishing Cat *P. viverrinus)*. *P. rubiginosus* is endemic to India, Sri Lanka and Nepal, with the larger part of its global population occurring in India^37^. This lineage originated during the Early Pliocene (4.59 Ma^32^), and *P. bengalensis* and *P. viverrinus* are sister species that diverged at the beginning of the Pleistocene (2.55 Ma^32^). Despite being closely related, the meagre information available on the ecology of the three species suggests that they have different ecological niches^34–36^. *P. viverrinus*, the largest of the three focal species, is a medium-sized felid weighing up to 16 kgs^38^ and strongly associated with mangroves, wetlands and reed beds^39^ . The species is morphologically adapted to hunting in water, with fish forming a prominent part of its diet along with small mammals and birds^38^. *P. bengalensis*, although distributed widely in India, is hypothesized to be restricted by high ambient temperatures exceeding 38 °C. It has not been reported from the hot central and western parts of the country, and genetic evidence suggests that the Western Ghats population is isolated from the rest of the species’ population^40^. The species does not occur in Sri Lanka^39^. Its diet includes rodents, other small mammals, birds, amphibians and reptiles^37^. Little is known about the ecology of *P. rubiginosus*, but preliminary data suggest it is primarily distributed through deciduous forests in India and its diet is believed to consist of rodents^38^. All three species are known to occur in proximity to human settlements^38^. *F. chaus* is a representative of the genus *Felis* that diverged from the *Prionailurus* lineage at 6.2 Ma^38^. It is a scrub and open habitat felid, strongly associated with water and very widely distributed in India^41^. It has benefited from irrigated agriculture that simulates its natural habitat and hence also occurs in proximity to human settlements^41^. It feeds largely on rodents and birds^41^.

### Species occurrences

We collected presence records for each species across the Indian subcontinent from published sources, online databases, and personal communications (Table SM1.2). Following previous recommendations^42^, records were pruned to keep only those contemporary with the time period of the environmental data used in analyses^43^. Thus, only records after 1990 were included. Moreover, duplicate records within the same cell (10 km resolution) were randomly removed using the gridSample function in the ‘dismo’ R-package^44^. For the period since 1990, after removing duplicate records (RD) within each cell, we obtained 75 records for *P. bengalensis*, 96 for *P. viverrinus,* 54 for *P. rubiginosus* and 87 for *F. chaus*. When applying a balanced design (RDbal, see details below), the number of records were 57 for *P. bengalensis*, 43 for *P. viverrinus,* 40 for *P. rubiginosus* and 62 for *F. chaus* (see distribution in Fig. SM1.2; full data in Table SM1.2). Although finer resolution environmental data is available, analyses were performed at 10 km resolution based on home-range estimates for the target species^37,38,45,46^, except for *P. rubiginous* for which estimates are not available. This resolution is therefore likely to minimize potential spatial autocorrelation while accounting for inaccuracies in the geographical positioning of records.

### Environmental data

Environmental predictors were chosen based on the species ecology and knowledge of their influence on carnivore occurrence^37,40,47–50^. Five environmental descriptors were considered: climate, topography, land cover, human disturbance and prey occurrence.

Topographic (elevation) and 19 bioclimatic variables were downloaded from the WorldClim data set^51,52^ (Table SM1.1). Each cell was also characterized based on land cover categories (1 km resolution) extracted from the Global Land Cover Map 2000 (including human settlements)^53^ to match the date of most occurrence records. The percentage of each land cover type within each cell was calculated by delineating each land cover and then summing the number of pixels within each 10 km^2^ cell using the aggregate function. Minor land cover classes were removed or reclassified together with other similar land cover types (Table SM1.1). Human population density estimates (2.5 arc-minutes resolution, ~ 5 km) and the Euclidean distance of every pixel to human disturbance proxies (e.g., distance to roads) were also included (Table SM1.1). To obtain the exact resolution, all rasters were resampled to the WorldClim rasters’ resolution (30 arc-second) using the bilinear method because the data is continuous^54^. All raster operations were performed in the ‘raster’ R-package^54^. Prey availability may also play an important role in small wild cat occurrence because they are obligate carnivores and have a strong preference for rodents, their staple prey^37,55,56^. Since prey selection has not been reported for the cat species studied here (although diet studies combined with data on prey availability are rare), we assumed that predation may depend more on other factors such as energetic cost, prey abundance and catchability^57^. We identified the most common rodent species preyed upon by carnivores in South-Southeast Asian forests through literature research (Table SM2.1) and used Maximum Entropy Modeling (Maxent)^58^ to estimate their environmental suitability across the Indian subcontinent (see details in SM2). We assumed that rodents of similar body size provide similar energetic benefits and have comparable probability of being caught, and therefore we pooled them into three body size groups (‘small’ ≤ 70 g, 70 g < ‘medium’ < 150 g; ‘large’ > 150 g) according to their weights (see Fig. SM2.1). We then used the output of these models as input to the niche models for the study species.

### Ecological niche models

#### Filtering strategies and bias files

Data clustering and bias can influence environmental niche model prediction^59,60^ and spatial data filtering has been recommended as an effective measure to decrease potential bias^59^. We first plotted occurrence records on the Indian subcontinent map and removed obviously erroneous georeferenced locations based on expert knowledge. Following recommendations from Kramer-Schadt et al.^61^ we prepared two differently filtered data sets. In the first one, all duplicate records within each cell (10 × 10 km) were removed (RD), while in the second one (RDbal) we randomly removed records until we obtained similar point densities (balanced design) across countries (Bangladesh, Bhutan, Nepal, Pakistan, Sri Lanka, India) in the study area (see Fig. SM1.2). All datasets were deposited in GitHub (https://github.com/andrepsilvadev/Prionailurus). Maxent assumes that species occurrence data are unbiased, independent samples from the distribution of the species (see MaxEnt tutorial—https://biodiversityinformatics.amnh.org/open_source/maxent/), and therefore does not consider uneven sampling effort. As a way of assessing possible bias in our data, we included two different types of bias files (BM01 and BM001) in our models^61^. In both files, a value of 1 was given for cells with occurrence records. For BM01, cells without samples/records were given a value of 0.1, indicating 10% of the sampling effort compared to cells with occurrence records, whereas for BM001 the same cells received a value of 0.01.

### MaxEnt modelling and single-descriptor models

Small felid occurrence was modelled using a maximum entropy-based machine learning algorithm that estimates the probability distribution for a species’ occurrence based on environmental constraints^58^. This algorithm was selected because it has been demonstrated to be among the most robust when using only presence records^62^ and for small sample sizes^63^. Due to the high number of explanatory variables used in the analyses, in order to be able to create models containing only the most important variables within each descriptor (see hybrid model section below), we first built separate models for each environmental descriptor with more than one variable (i.e., climate, land cover, human disturbance and prey occurrence). Within each descriptor, for highly correlated (r > 0.65) variables, we omitted one from the pair, retaining those of easier biological interpretation or representing extreme environmental conditions (e.g. temperature of the warmest month) (Table SM1.3). All models were run in Maxent v3.3.3 k^58^ using 70% of the records as training data (random partitioning) with 10,000 background points^64,65^. The remaining 30% of the occurrence records were used for model evaluation avoiding inflating the ‘area under the receiving operating characteristic curve’ (AUC) values by using the same locations for training and testing. For each model, we ran 10 replicates with 5000 iterations to allow for model convergence, and used the subsample strategy (i.e. the presence points are repeatedly split into random training and testing subsets) as a form of replication in order to maximize the number of locations used for model testing. We used Maxent’s raw output, which expresses the relative probability that a cell contains a presence record, and consequently cells with relatively low raw values may still have a high absolute probability of presence, albeit lower than other cells. This output avoids default assumptions on the probability of presence at locations with ‘‘average’ conditions for the species, which are necessary for the common logistic output^66,67^. Following previous proposals^68^, we used species-specific model tuning to find the optimal settings for Maxent models (see full details in SM1), totalling 24 candidate models per species.

#### Model selection and relative variable importance

To find the best models in each set we first selected a subset of models with ‘area under a relative operating characteristic curve’^69^ for the test data (AUCtest) > 0.7. Then, among those, the ones with the lowest test omission rates (false negatives) and AUCdiff (AUCtrain–AUCtest), a measure of overfitting^70^, were selected. Three threshold metrics (MTP—minimum training presence, P10—10th percentile training presence, ETS—equal test sensitivity and specificity) were used to measure omission rates^61^. Finally, to measure spatial overlap between best model predictions for each species we calculated Schoener’s overlap metric (D)^71^ and the modified Hellinger metric (I)^72^ using the “calc.niche.overlap” function from the ‘ENMeval’ R-package^73^. Models with AUCtest values between 0.7 and 0.9 were considered to have useful accuracy, and models with AUCtest > 0.9 were considered to have high accuracy^74^. Following model selection, we calculated variable permutation importance within each environmental descriptor (for details on how variable importance was calculated see SM1). To aid our decision regarding the most important variables within each descriptor, we cross-checked their importance for model gain and AUCtest for each variable (see Figs. SM1.2–SM1.10).

#### Hybrid model

A species’ environmental niche is likely to be influenced by more than one environmental descriptor. In order to produce more realistic estimates of the species environmental niches, we built a hybrid model for each species. These models included only the most important climatic, topographic, land cover, human disturbance and prey occurrence variables for each species, as assessed through the variable’s relative importance in the single-descriptor models. This strategy reduces the number of variables included in the models, thereby decreasing the potential for overfitting. Maximum correlation between variables included in the same hybrid model was 0.64 (Table SM1.3). To understand the relative importance of climatic predictors compared to other abiotic and biotic predictors, we also tuned models and estimated the relative importance of each variable included in the hybrid model as we did for the single-descriptor models. Finally, to assess if climate-only models were good surrogates for more complex models incorporating non-climatic information, we calculated spatial overlap between predictions for current time from climate-only models and from the best hybrid models. Final hybrid models used to calculate niche overlap and protected area coverage were also evaluated using specificity and the symmetric extremal dependence index (SEDI) based on the P10 threshold^75^.

### Spatial overlap and tests of niche conservatism

To test potential niche conservatism among the study species we followed available quantitative approaches to niche evolution^72^. We used the continuous raw predictions from the best hybrid models to calculate Schoener’s D and the modified Hellinger metric. In addition, we calculated these metrics in environmental space using the original species occurrence records, to account for possible biases originating from the extent and distribution of environmental gradients in geographic space^76^. Following the spatial overlap analysis, we used niche equivalency and similarity tests to assess niche divergence in environmental space. Following Broennimann et al.^76^, in the niche equivalency test all occurrences were pooled and then randomly split into two datasets, each with the same original number of occurrences. This process was repeated *n* times and the distribution of simulated values was compared to the observed overlap value. The null hypothesis of niche equivalency cannot be rejected if the observed value falls within the simulated distribution. On the other hand, given two species (X and Y), the niche similarity test evaluates similarity between cells where species X occurs and a random set of cells, with sample size equal to that for species Y, within the study area of Y^72^. Thus, in this test, for each pair of species, two comparisons are carried out, one between species X and the null density of occurrence for species Y, and vice versa. Each comparison is repeated *n* times to generate a distribution of simulated values against which the observed value is compared. All tests were carried out in the R-package ‘ecospat’^77^ using 1000 replications.

### Protected area coverage

To evaluate the agreement between species’ environmental affinities and current spatial conservation strategies, here measured as protected area coverage, we applied presence thresholds (MTP, P10, ETS) to transform species environmental suitability predictions from the hybrid models into binary maps of predicted species presence. Next, we extracted shapefiles of terrestrial protected areas from the World Database on Protected Areas^78^, excluding those with a “Proposed” or “Not Reported” status, and superimposed them over the binary predictions of species occurrence to calculate the mean proportion of the species’ binary predictions covered by protected areas. All analyses and maps were built in R 3.4.3^79^.

### Species climatic suitability through time

As we detected different important variables for each species under current environmental conditions (see results below), we additionally estimated species response to environmental change dynamics through time. We projected the best climate-only models onto projections of past and future climate, and then calculated the proportion of the Indian subcontinent with suitable climate for each species at particular time intervals. We did this only with climatic data because estimates for other environmental variables are highly uncertain or unknown for past and future conditions. Moreover, during initial analyses (data not shown) we detected that climatic predictors were overall among the most, if not the most, important predictors for each species at the scale of analysis in this study.

To represent past climate conditions we used the Otto-Bliesner’s simulation model^80^ based on a general circulation model CCSM2 for the period ~ 140 to 120 ka of the last interglacial (LIG), which predicts particularly warm conditions during the warmest quarter for the Indian subcontinent (Fig. SM1.1). For the remaining time periods we used climate predictions based on a Community Climate System Model (CCSM) but we also included climate predictions from other General Circulation Models (GCMs) to account for uncertainty in climate model projections. Specifically, for the mid-Holocene (MH) and Last Glacial Maximum (LGM) we used the CCSM4, MIROC-ESM, and MPI-ESM-P models, following previous literature^81,82^. For future conditions (year 2070), we used models with satisfactory performance or reduced bias^83^. Of these, the ones downscaled, calibrated, and available in the WorldClim database^51^, were selected for projections. The General Circulation Models (GCMs) used were GFDL-CM3, MPI-ESM-LR, bcc-csm1-1-m, CCSM4, CNRM-CM5, HadGEM2-ES, and IPSL-CM5A-LR. For projections of future climate, two emission scenarios were considered: the Representative Concentration Pathway (RCP) 4.5, an optimistic scenario where emissions peak around 2040, and the RCP 8.5, a pessimistic scenario where emissions continue to rise through the twenty-first century. Finally, following recommendations by Nogués-Bravo^84^*,* we also identified geographical areas in which past and future non-analogous climates are out of the calibration range (Figs. SM1.14, SM1.15), as this may increase the chance of naive projections and therefore should be taken into account when interpreting results (Figs. SM1.14, SM1.15).

## Results

### Variable importance

**Figure 1:**
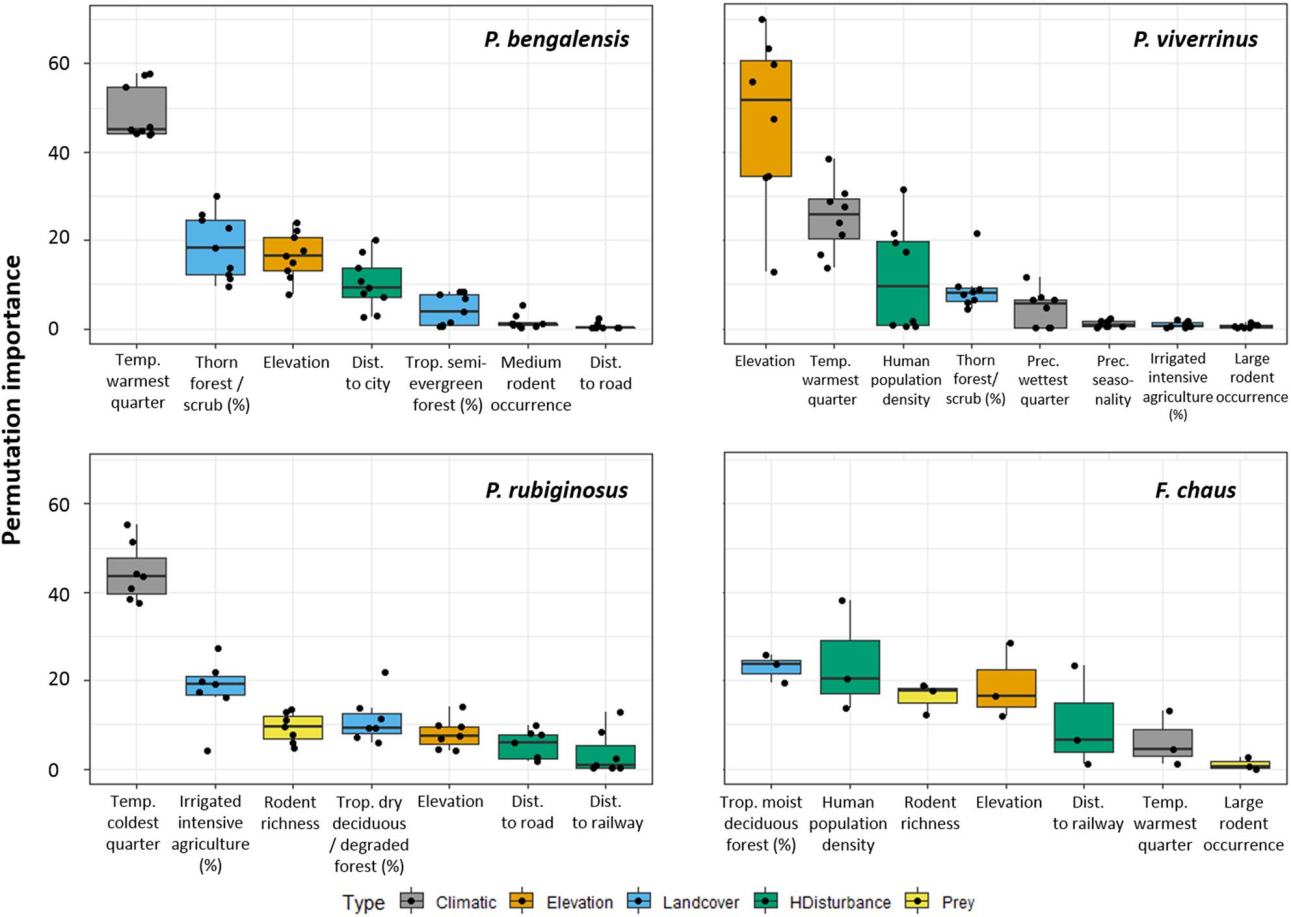
Climate, land cover and topography tended to be, overall, the most important factors explaining species occurrence. Notably, the most important variables were species-specific. Variables (see detailed description in Table SM1) are ordered from highest to lowest permutation importance.

The hybrid models indicated that climate, land cover and topography were the main determinants of species occurrence (Fig. 1). However, variables with the highest permutation importance within each single-descriptor model (see full details in supplementary material—SM1), and therefore included in the hybrid models, were different among species (Fig. 1). *P. bengalensis* was negatively associated with high temperature in the warmest quarter (Bio 10), and *P. rubiginosus* was positively associated with warmer temperatures in the coldest quarter (Bio 11) (Fig. SM1.32). *P. viverrinus* was mainly associated with lower elevation areas (Fig. SM1.33), whereas there was no single most important variable for *F. chaus*. Variables associated with human-modified land cover or human presence also had considerable permutation importance, namely a negative association with irrigated intensive agriculture (*P. rubiginosus*) and positive association with human-related landscape features (*P. bengalensis* and *P. rubiginosus*) and human population density (*P. viverrinus* and *F. chaus*) (Fig. SM1.34). The combinations of environmental variables included in the hybrid models were species-specific (Fig. 1). Overall, the best hybrid models had low omission rates (threshold; mean ± sd) for *P. bengalensis* (P10; 0.09 ± 0.02), but moderate for *P. viverrinus* (P10; 0.12 ± 0.03), *P. rubiginosus* (P10; 0.13 ± 0.06) and *F. chaus* (P10; 0.14 ± 0.04). Model predictive accuracy (mean AUCtest ± sd) was high for *P. bengalensis* (0.90 ± 0.02), *P. viverrinus* (0.93 ± 0.02) and useful for *P. rubiginosus* (0.87 ± 0.02) and *F. chaus* (0.81 ± 0.01). Overall SEDI confirmed model usefulness for *P. bengalensis* (0.78 ± 0.02), *P. viverrinus* (0.88 ± 0.01), *P. rubiginosus* (0.76 ± 0.03) and *F. chaus* (0.55 ± 0.06). The best hybrid models mainly included L, LQ or auto features, but different regularization multipliers (RM1–RM2) and sampling strategies. Full details of hybrid models can be found in supplementary material (Tables SM1.6–SM1.8).

### Spatial overlap and niche conservatism tests

**Figure 2:**
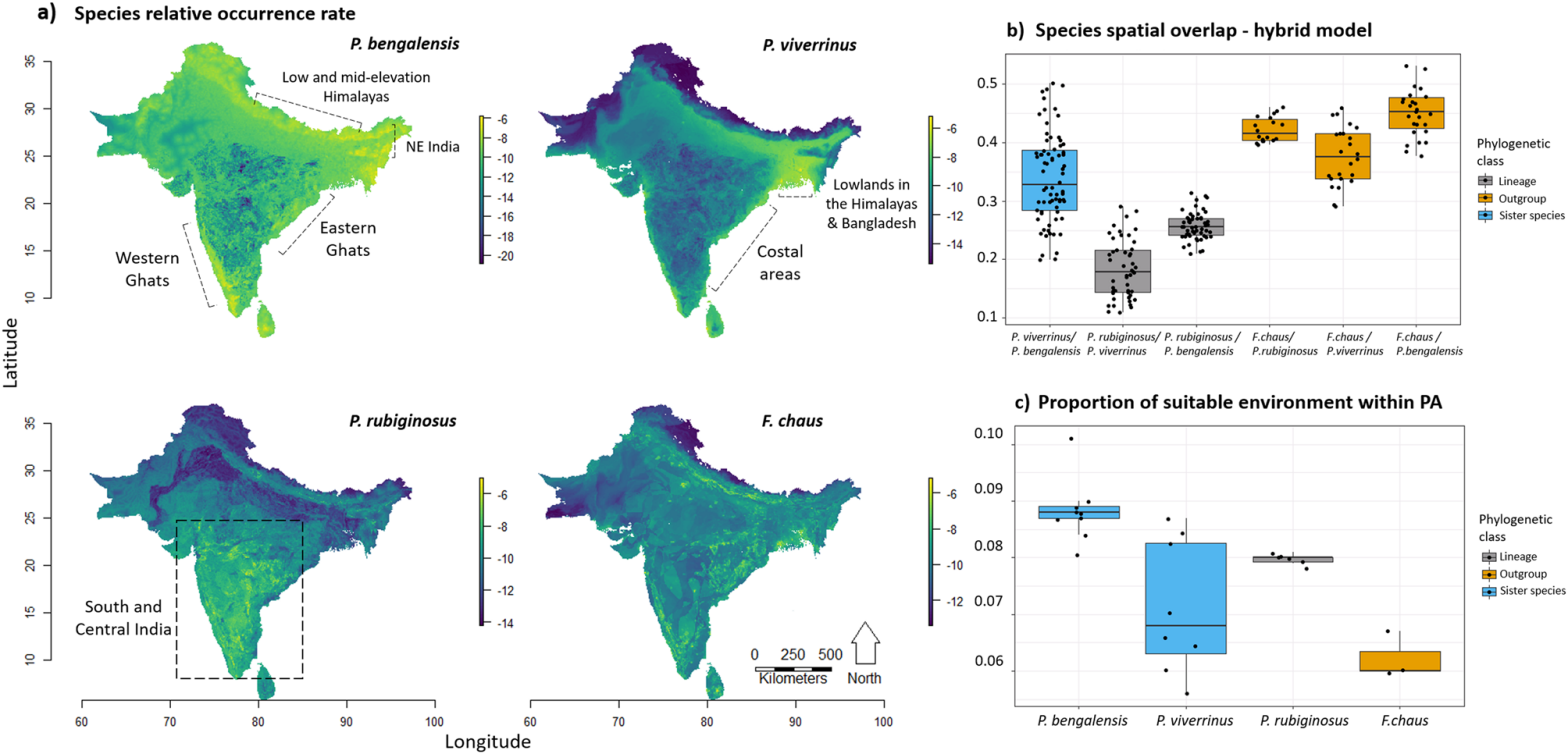
(**a**) Species relative occurrence rates (ROR), displayed as log(ROR), in the Indian Subcontinent as predicted by the best hybrid models; (**b**) Species spatial overlap (Schoener’s D). Similar patterns were found with the modified Hellinger metric I (Fig. SM1.37); (**c**) Protected area coverage of species potential occurrence (calculated based on binary maps—P10 threshold); Black filled dots correspond to predictions for the best models. Similar patterns were found with protected area coverage calculated using different thresholds (MTP, ETS) (Fig. SM1.40).

Hybrid model predictions showed that areas with high relative occurrence rate tend to differ between *Prionailurus* species. *P. bengalensis* had higher occurrence rate predicted for Northeast India and along the mid and low elevations of the Himalayas and Western Ghats, as well as for Sri Lanka (where the species is not known to occur) (Fig. 2a). The highest rate of occurrence of *P.* viverrinus was, in turn, predicted for low elevations along the Himalayas and, in particular, Bangladesh, but also in Sri Lanka (Fig. 2a). In contrast, the most suitable areas for *P. rubiginosus* were estimated to extend across south and central India. For *F. chaus* there were a few particular areas with higher occurrence rates along southeastern India, Eastern Ghats and the Himalayan lowlands, but optimal conditions seem to be widespread throughout the subcontinent (Fig. 2a). Although the sister species *P. bengalensis* and *P. viverrinus* had a higher estimate of overlap (Schoener’s D: 0.340 ± 0.077), this was not considerably greater than the estimates obtained between *P. rubiginosus* and *P. bengalensis* (Schoener’s D: 0.257 ± 0.024) and between *P. rubiginosus* and *P. viverrinus* (Schoener’s D: 0.184 ± 0.050) (Fig. 2b). A tendency for greater spatial overlap between *Prionailurus* species and the outgroup was observed, but still within the range of the highest overlap estimates among *Prionailurus* species (Schoener’s D for *F. chaus* vs. *P. viverrinus*: 0.377 ± 0.049; *F. chaus* vs *P. rubiginosus*: 0.422 ± 0.021; *F. chaus* vs *P. bengalensis*: 0.451 ± 0.041) (Fig. 2b). When considering full environmental space (defined by all variables included in hybrid models), niche equivalency could not be rejected (*P* = 0.325) between *P. viverrinus* and *P. rubiginosus* and between *P. viverrinus* and *F. chaus* (Fig. SM1.38). Niche similarity between species could also not be rejected (*P* = 0.170) in any of the tests (Figs. SM1.38, SM1.39).

### Protected area coverage

Protected area coverage was overall low (min = 0.056; max = 0.105) and heterogeneous among species (Fig. 2c). Lower coverage was found for *F. chaus* (0.062 ± 0.004) and *P. viverrinus* (0.071 ± 0.012), and the highest coverage was found for *P. bengalensis* (0.088 ± 0.006). *P. rubiginosus* had intermediate coverage (0.080 ± 0.001). Patterns of protected area coverage were similar across the different thresholds tested, with the most notable differences being that the estimates were slightly lower for *P. viverrinus* and higher for *F. chaus* when using the MTP threshold (Table SM1.10, Fig. SM1.40).

### Species climatic suitability through time

**Figure 3:**
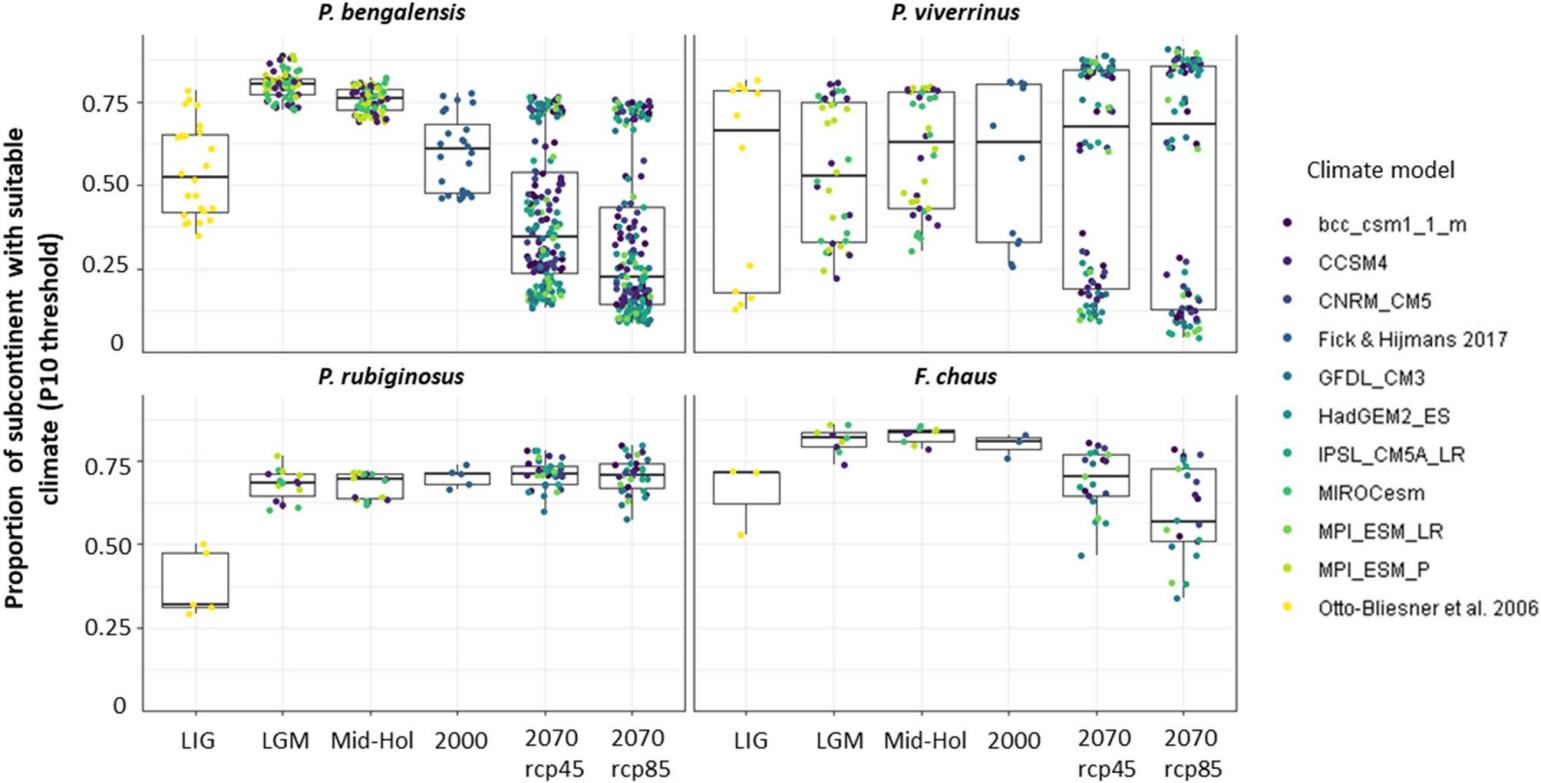
Species climatic suitability (P10 threshold) in the Indian subcontinent since the last interglacial (LIG; ~ 140 to 120 ka) up to 2070 (for an optimistic scenario, RCP 45, and a business-as-usual scenario, RCP 8.5). Colors indicate the climate models used for past and future projections. LGM—Last Glacial Maximum (~ 22 ka); MH—Mid-Holocene (~ 6 ka); Current time (1950–2000). Spatial representation of mean raw predictions for each time period is provided in SM1 (Fig. SM1.18–SM1.21).

The importance of particular climatic variables for each species led to species-specific trends of climatic suitability through time (Fig. 3). Future (year 2070) climatic suitability is estimated to decrease for *P. bengalensis* and *F. chaus*, compared to current time, under both RCP scenarios. Climatic suitability for *P. bengalensis* appears to have been declining considerably since at least the mid-Holocene (~ 6 ka), but the negative trend will accelerate given the predicted human-induced climate change. For *P. rubiginosus*, models estimated an increase in climatic suitability from the last interglacial (~ 140 to 120 ka) to the Last Glacial Maximum (~ 22 ka), and stability since then up to 2070. Predictions for *P. viverrinus* were less clear as they varied according to the best models considered. While most models inferred a slight increase in median climatic suitability since the LGM, a few models estimated a drop in climatic suitability since the mid-Holocene, continuing in the forecasted future. Estimated trends based on MTP and ETS thresholds showed similar patterns to those using P10 thresholds (Figs. SM1.16, SM1.17). Species-specific responses to climatic factors also explain the different predicted climate refugia areas among the studied species, with refugia only overlapping potentially in the lowlands of Bangladesh and West Bengal (Fig. SM1.23). The considerable overlap between the predictions of climate-only and hybrid models for the present time (Fig. SM1.27), with the exception of a larger difference for *P. viverrinus*, suggests that climate-only model projections for the past and future may closely approximate more complex models.

## Discussion

We show that *Prionailurus* species have less or similar spatial overlap with each other than in relation to an outgroup species. Also, within *Prionailurus* there was no greater spatial overlap between sister species, with niche equivalency rejected in environmental space. Therefore, we found no support for phylogenetic niche conservatism within *Prionailurus*. Instead, particular environmental niche characteristics result in species-specific environmental responses, translating into potentially heterogeneous responses to future climate change. Moreover, current areas of high environmental suitability for the different species are not equally, and adequately, covered by the existing protected area network. In this scenario multispecies spatial conservation planning becomes challenging.

### Insights on species responses to global environmental change

Our study highlights that climatic suitability for closely related species can be species-specific. This increases the body of evidence showing lack of phylogenetic niche conservatism in mammalian species, and reinforces the importance of understanding deep-time species history and speciation mechanisms before assuming common responses and conservation strategies delineation^24,85^. In particular, the patterns found match with a speciation process related to environmental divergence, similar to that found in other tropical mammals^22,23^. Such process would have required a potential niche shift from a common ancestor, hence suggesting that future response to environmental change may be determined by other key ecological processes such as dispersal and biotic interactions, potentially with species with similar niche but not necessarily closely related. This stresses the necessity of not focusing only on the search for common conservation areas but also on maintaining connectivity and community structure.

Further, assuming abiotic niche stability, our results apparently suggest that endothermic species with a tropically restricted geographic range (*P. rubiginosus* and *P. viverrinus*) may see their occurrence areas maintained or increased with global warming, whereas more thermal generalist species (*P. bengalensis* and *F. chaus*) may not be able to cope well with higher temperatures in the tropical parts of their ranges (Fig. 3). This is in contrast to predictions for temperate regions, where global warming is predicted to have a negative effect on species with a preference for cold climate, and a positive effect on more thermal generalist species^86–89^. This is also contrary to predictions for tropical ectotherms, which have been suggested to be particularly vulnerable to climate warming because of their physiological sensitivity to temperature changes^90,91^.

In addition we found indications that occurrence contractions may have begun before anthropogenic impacts although these may have contributed to the trend. For example, it was estimated that *P. bengalensis’* climatic suitability may have been decreasing since the LGM. Although the importance of natural dynamics of climatic suitability through time for explaining current biodiversity patterns has been long known^84,92^, and there is recent work facilitating the understanding of such dynamics^93,94^, our study highlights the need for ways to disentangle natural species’ suitability dynamics and human-driven species distribution changes, so that both can be taken into account when predicting species vulnerability to global change.

### Implications for Prionailurus cats’ conservation

*Prionailurus* species are unequally covered by the current protected area network. Protected areas are often located in forested regions (generally assumed to be associated with higher species richness), in habitats used by charismatic species, such as tiger reserves in India, or in places less suitable for humans due to rough topography or low economic potential^95–97^. This is perhaps the reason why *P. bengalensis,* which is associated with tropical semi-evergreen forests and can inhabit higher altitudes, has greater coverage of its range by protected areas than the other species. It is important to note that the metric used to measure protected area coverage is sensitive to the extent of the species’ geographic range, so that species with a larger distribution area, such as *F. chaus*, tend to have a smaller proportion of it covered by protected areas, although in absolute terms they may have more geographic range covered. Overall, the low estimates of protected area coverage (below 10%) indicate important gaps in the current protected area network in the Indian subcontinent. Moreover, it is important to note that the coarse grain size used in the analyses can lead to overprediction of presence area (this overprediction being variable between species). This is particularly possible in heterogeneous landscapes^98,99^ such as the Himalayas or the Western Ghats. Therefore, the low overall estimate of protected area coverage may still, in fact, be an overestimation.

This study highlights climatic change and human-driven land conversion as the two main macroscale factors negatively influencing the occurrence of *Prionailurus* cats in the Indian subcontinent. Climatic suitability shrinkage is particularly evident for *P. bengalensis*. For *P. bengalensis*, increased suitability from the LIG to LGM is in agreement to climatic suitability expansion found for forest rodent species during the same period in Southeast Asia^100^. Also, a tendency for higher, but overlapping, suitable climatic area during the LGM compared to the mid Holocene resembles the pattern described for other Indochinese mammals^101^. Moreover, considerable genetic differentiation has been found between northern and southern Indian Leopard Cat populations^40^, which may have been exacerbated by late Holocene environmental events, as predicted by the drop in climatic suitability from the mid Holocene to the present time. While climatic suitability across the Himalayas appears to have been stable over time (Fig. SM1.18), it is much more dynamic in south and central India, with an expected future decline in the Eastern and Western Ghats. In fact, the models predict a complete loss of climatic suitability in the Eastern Ghats under the most pessimistic RCP scenario; on the other hand, in the Western Ghats the complete loss of climatic suitability is not expected under any RCP scenario (Fig. SM1.18). The Western Ghats is actually a climate refuge (Fig. SM1.23) where the populations of *P. bengalensis* harbor unique genetic diversity^40,102^ and the species is commonly detected^40,49,103^. Observations in the Eastern Ghats are, in contrast, much sparser (but see for instance^87^). Therefore, mitigating current anthropogenic threats to the populations of *P. bengalensis* in the Western Ghats should be a priority to avoid a cumulative effect of human impacts and natural oscillations of climatic suitability. This strategy has the advantage of maintaining a greater evolutionary potential in the species and allows the possibility of re-expansion during periods of climate cooling, as estimated to have occurred after the last interglacial (Fig. SM1.18). It is important to note that *F. chaus*, for which a demographic expansion has been estimated at 271–166 ka^40^, a time interval that includes the interglacials of Marine Isotope Stage (MIS) 7^104^, and is now a widespread species in the Indian subcontinent, may not be immune to future climatic change, since disconnection between climatic suitability patches in the Western Ghats and central-north India is predicted (Fig. SM1.21), a pattern similar to that currently inferred for *P. bengalensis*.

For *P. viverrinus*, the consequences of climate change are unclear, since the selected best climate-only models showed considerable variation in predicted future climate suitability (Fig. 3). Such variation could arise from variation in the climate models used to represent past and future climate scenarios, but Maxent models projected into the same climate scenarios still show varied predictions (Fig. 3), suggesting that prediction variation originates from model fitting instead. Consequently, we cannot exclude a potential decrease in the climatic suitability for the species under the climate change scenarios used here. However, a preliminary Cyt *b* study (Shomita Mukherjee, unpublished data) inferred connectivity between Northern and Eastern India populations that is compatible with stable climatic suitability over time. Our averaged predictions over time are also broadly coherent with past population dynamics inferred for their Indochinese range from mitochondrial DNA^105^. Given this uncertainty, we recommend conservation focus on areas with stable climatic suitability over time, namely the lowlands of Bangladesh and West Bengal (Fig. SM1.23). We also noted a disagreement between predicted suitable environment from hybrid models (i.e., the fundamental niche) and the highly fragmented distribution areas of the species^35^. This may be an indication that unconsidered factors, such as illegal killing^106^ and road kills^107^, may currently restrict the occurrence of the species. Unfortunately, *P. viverrinus* is likely benefiting little from protected land (showed the lowest protected area coverage—Fig. 2C). We therefore suggest increasing protected habitat for the species and echo the need for urgent local conservation actions^35^, especially in the lowlands of Bangladesh and West Bengal, the climate refugia identified in 90% of the models.

*P. rubiginosus* was considered an endemic species of south India and Sri Lanka, but has recently been detected in north India and Nepal^108–110^. Although this could be a consequence of the recent increase in camera-trap surveys, a phenomenon that is likely confounding estimation of population trends for other species in India^111^, it may be the result of natural expansion of *P. rubiginosus* into previously cooler areas due to climate warming. The Himalayan lowlands were not identified as climatic refugia (Fig. SM1.23) but may have suitable climate in the future (Fig. SM1.20). We therefore recommend that population viability in climatic refugia (south India and Sri Lanka, Fig. SM1.23) should be ensured, and that exploratory surveys should be conducted throughout the Himalayan lowlands to investigate a potential northward expansion of the species.

Although we have confidence in our models, we advise caution regarding predictions for desert areas in Pakistan and India, as well as for central India, under more extreme future climate conditions (RCP8.5), as the temperature of the warmest quarter (Bio 10) can exceed the range used for model calibration (Fig. SM1.11). The same should be taken into account when considering the influence of the temperature of the coldest quarter (Bio 11) in south India (Fig. SM1.12).

It should also be stressed that other drivers of environmental change (e.g., land use) can exacerbate or mitigate changes in climatic suitability, and possible interactive effects have recently been described^112,113^. For example, the negative relationship of *P. rubiginosus* with intensive irrigated agriculture suggests that North India´s cropland belt around the Ganga basin is a major unsuitable area separating suitable environment in south India and suitable habitat patches in the Himalayan lowlands (Fig. 2A). The expected increase in cropland^114^ is therefore likely to further negatively impact the species. The relationship of the species with human disturbance was not, however, straightforward, as they all showed a high probability of occurrence close to human structures or with increasing human population density, although human disturbance variables tended to be of low importance. This positive relationship may be an artifact due to biased data regarding human presence (despite corrections with bias files) or may correspond to species’ use of human-modified environments, due for instance to greater resource availability (e.g., prey abundance), as we detected association of some rodent genera with human settlements and population density (Fig. SM2.3). The intricate link between human and rodent prey presence, together with the importance of climatic factors for most of the genera of rodent prey (Fig. SM2.3) and the species-specific responses of each small cat species, point to complex responses to global change.

## Supplementary information

Supplementary Information 1.

Supplementary Table 1.

Supplementary Information 2.

Supplementary Table 2.
