# Supplementary material for: Closely related species show species-specific environmental responses and different spatial conservation needs: Prionailurus cats in the Indian subcontinent
Source: Sci Rep. 2020 Oct 30;10:18705. doi: 10.1038/s41598-020-74684-8 (PMC7599212; doi:10.1038/s41598-020-74684-8)
Supplement: Supplementary file 1 — Supplementary Information 1. [file 41598_2020_74684_MOESM1_ESM.docx]

**SM1 - supplementary material for**

**Title**: Closely related species show species-specific environmental responses and different spatial conservation needs: *Prionailurus* cats in the Indian subcontinent

**Authors**: André P. Silva, Shomita Mukherjee, Uma Ramakrishnan, Carlos Fernandes, Mats Björklund

**Single descriptor model tuning**

Following previous recommendations [^1^](https://paperpile.com/c/fJYCIu/KRsQo), we used species-specific model tuning to find the optimal settings for MaxEnt models. Since species response to environmental predictors tends to be complex, MaxEnt can create an expanded set of transformations of the original covariates (termed features) to fit nonlinear functions [^2^](https://paperpile.com/c/fJYCIu/kYQ0I). Several transformations are available: linear (L), quadratic (Q), product (P), threshold (T), hinge (H); see detailed description [^3^](https://paperpile.com/c/fJYCIu/9zWOi). Model complexity can increase considerably with the use of several features. Hence, for each species, we built simpler models using the same feature subsets (L, LQ, H and LQH) previously used for data-poor species [^1^](https://paperpile.com/c/fJYCIu/KRsQo). The careful tuning of the regularization multiplier (RM) when conducting Maxent modeling has also been reported to be essential to avoid model overfitting [^4^](https://paperpile.com/c/fJYCIu/qtqec). Therefore, we ran models with different RM values (1, 1.5, 2) to identify the best RM for the final predictive models. The RM values tested were based on available literature [^4^](https://paperpile.com/c/fJYCIu/qtqec), who reported possible overfitting for models with RM < 1, while models with RM = 2 generally correspond to broad environmental classes, like vegetation types, where the species can occur, and possible underfitting can occur for models with RM > 3. RM values were tested for each spatially filtered data set, bias file and feature set, totalling 24 candidate models per species.

***Variable relative importance***

Following model selection, we calculated variable permutation importance within each environmental descriptor. To calculate permutation importance, the values of each variable on training presence and background data are randomly permuted in turn [^5^](https://paperpile.com/c/fJYCIu/MlgLm). The model is then reevaluated on the permuted data, and the resulting drop in training AUC is given normalized to percentages. This approach allowed us to identify the most important climatic, land cover, human disturbance and prey predictors for each species. When several best models were selected, we took the average permutation importance for each model (calculated across the 10 runs) and looked at the median, 25th and 75th percentiles of average variable permutation importance from the best models. To aid our decision regarding the most important variables within each descriptor, we cross-checked their importance for model gain and AUCtest for each variable (see Supplementary Fig. SM1.3 - 1.10).

**
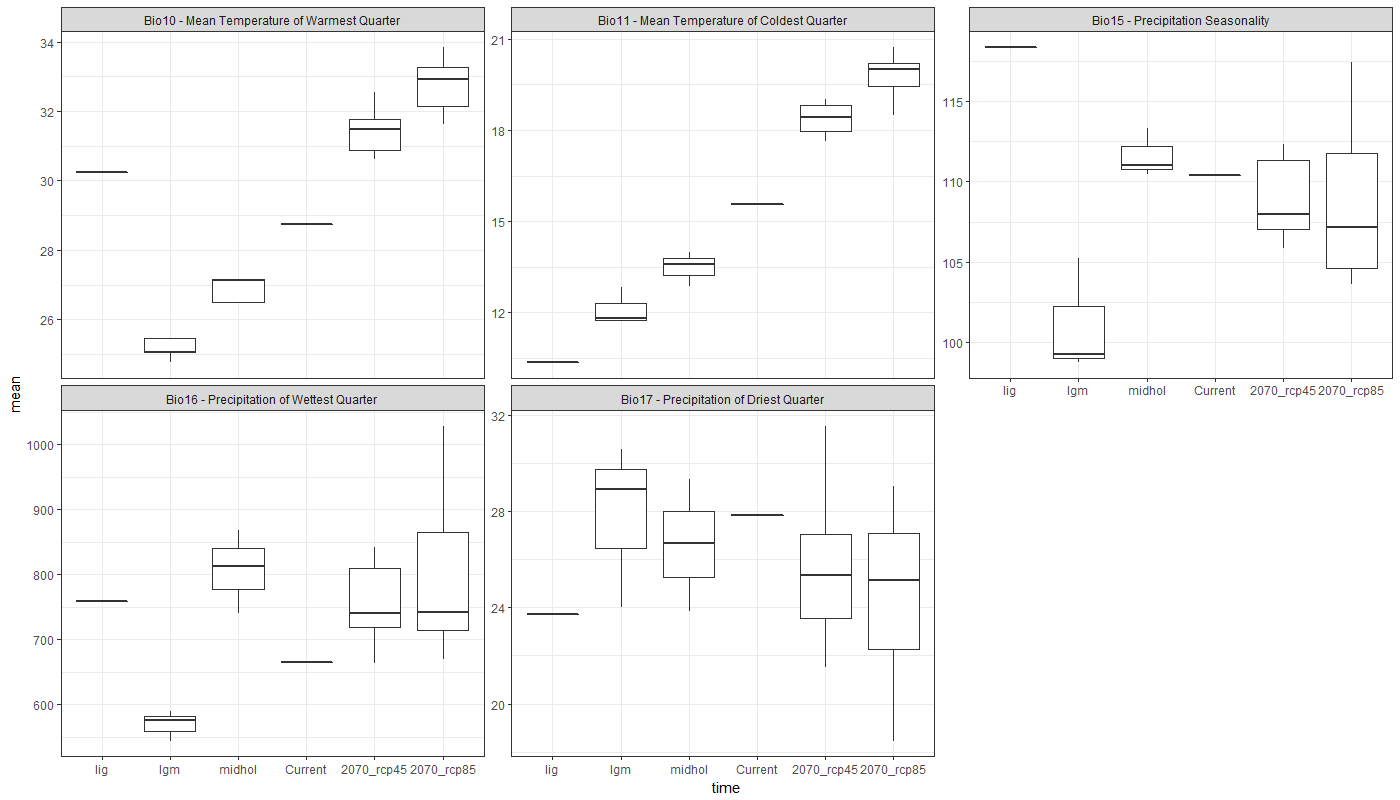
**

**Figure SM1.1**- Predictions for temperature (tºc) and precipitation (mm) (only main climatic variables included in the hybrid ENMs) in the Indian subcontinent across time according to global climate models selected for this study (see Methods). Main climatic changes for 2070 are the predicted increase of mean temperature for both the warmest and coldest quarters of the year.


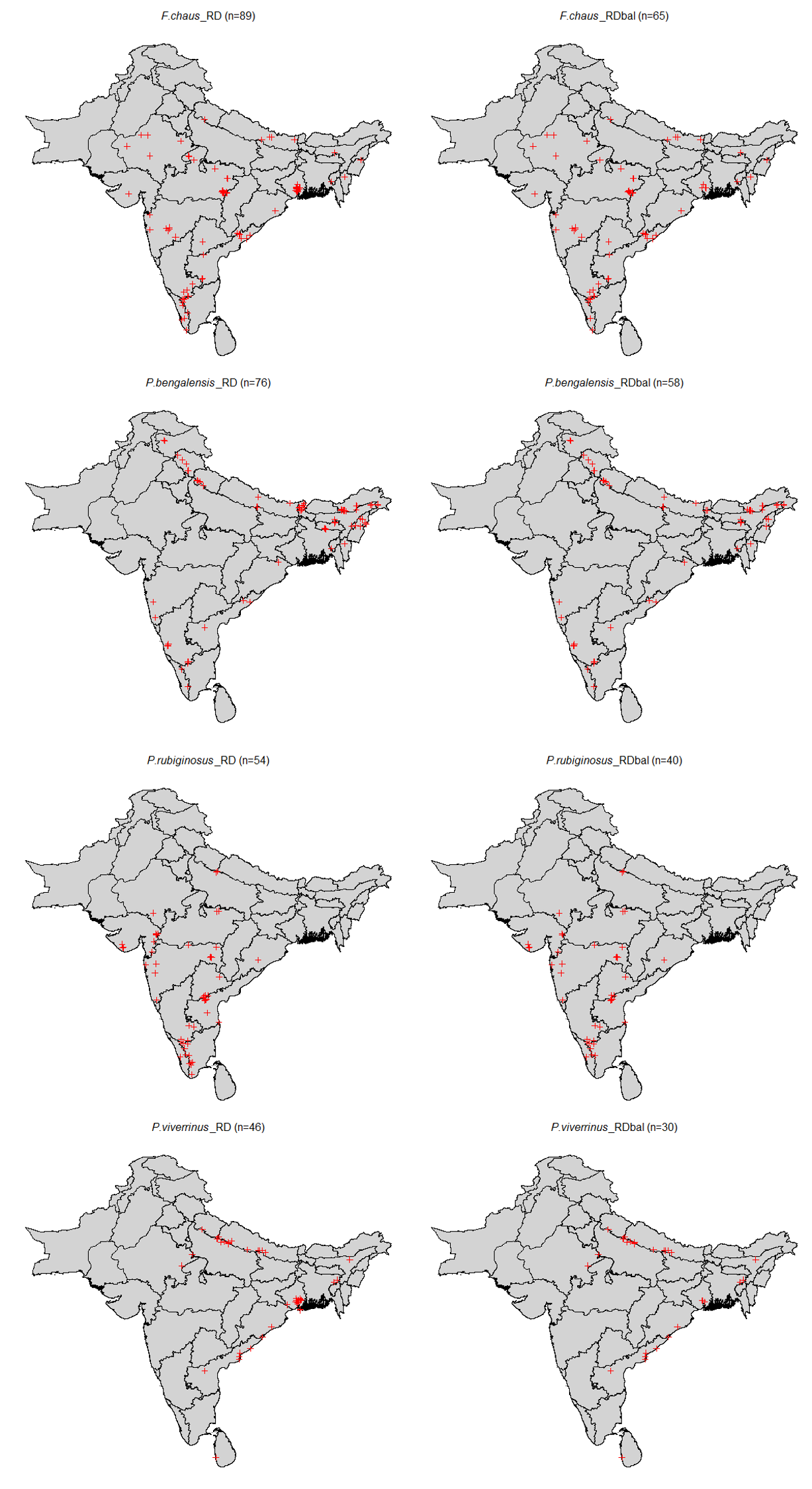


**Figure SM1.2** - Occurrence records used for modeling species occurrence across the Indian subcontinent after removing duplicate locations (RD) within the same cell and after randomly removing locations from over-sampled administrative areas, until achieving average point density for sampled areas (RDbal).

**Results**

*Single-descriptor models*

For the climate-only models, the mean temperature of the warmest quarter (Bio 10) was the highest contributing variable for *P. bengalensis* and *F. chaus* and the second most important for *P. rubiginosus*, for which the variable with the highest permutation importance was mean temperature of the coldest quarter (Bio 11). For *P. viverrinus*, however, precipitation seasonality (Bio 15) and precipitation of the wettest quarter (Bio 16) contributed the most, together with Bio 10 (Fig. SM1.3). Regarding the land cover-only models, thorn forest / scrub (TFS) (*P. bengalensis* and *P. viverrinus*) or irrigated intensive agriculture (IIA) (*P. rubiginosus*) had the highest permutation importance for *Prionailurus* species. *F. chaus* occurrence was mainly explained by tropical moist deciduous forest (TMD). Looking at mean test gain and AUCtest, additional variables appeared as potentially important for explaining species occurrence, in particular tropical semi-evergreen forest (TS) (*P. bengalensis*), irrigated intensive agriculture (*P. viverrinus*), and tropical dry deciduous and degraded forest (TDD) (*F. chaus*) (Fig. SM1.5). For the human-disturbance models, distance to nearest primary or secondary road (DistRds) was important for *P. bengalensis* and *P. rubiginosus*, whereas human population density (PopDens) had high importance for *P. viverrinus* and *F. chaus*. Distance to railways (DistRail) also contributed to explain the occurrence of *P. rubiginosus* and *F. chaus* (Fig. SM1.7). Finally, concerning the prey occurrence models, rodent richness (Rodentia SR) contributed to explain *P. rubiginosus* and *F. chaus* occurrence, medium-sized rodent occurrence (MRO) (70g < weight < 150g) contributed to explain *P. bengalensis* occurrence, and Larger-bodied rodents (LRO) (>150 g) contributed to explain the occurrence of *P. viverrinus* and *F. chaus* (Fig. SM1.9). Overall, model accuracy (mean AUCtest ± sd) for test data was high for climate models (0.87 ± 0.06), land cover models (0.84 ± 0.07) and prey models (0.83 ± 0.05), and useful for human disturbance models (0.77 ± 0.08).

**
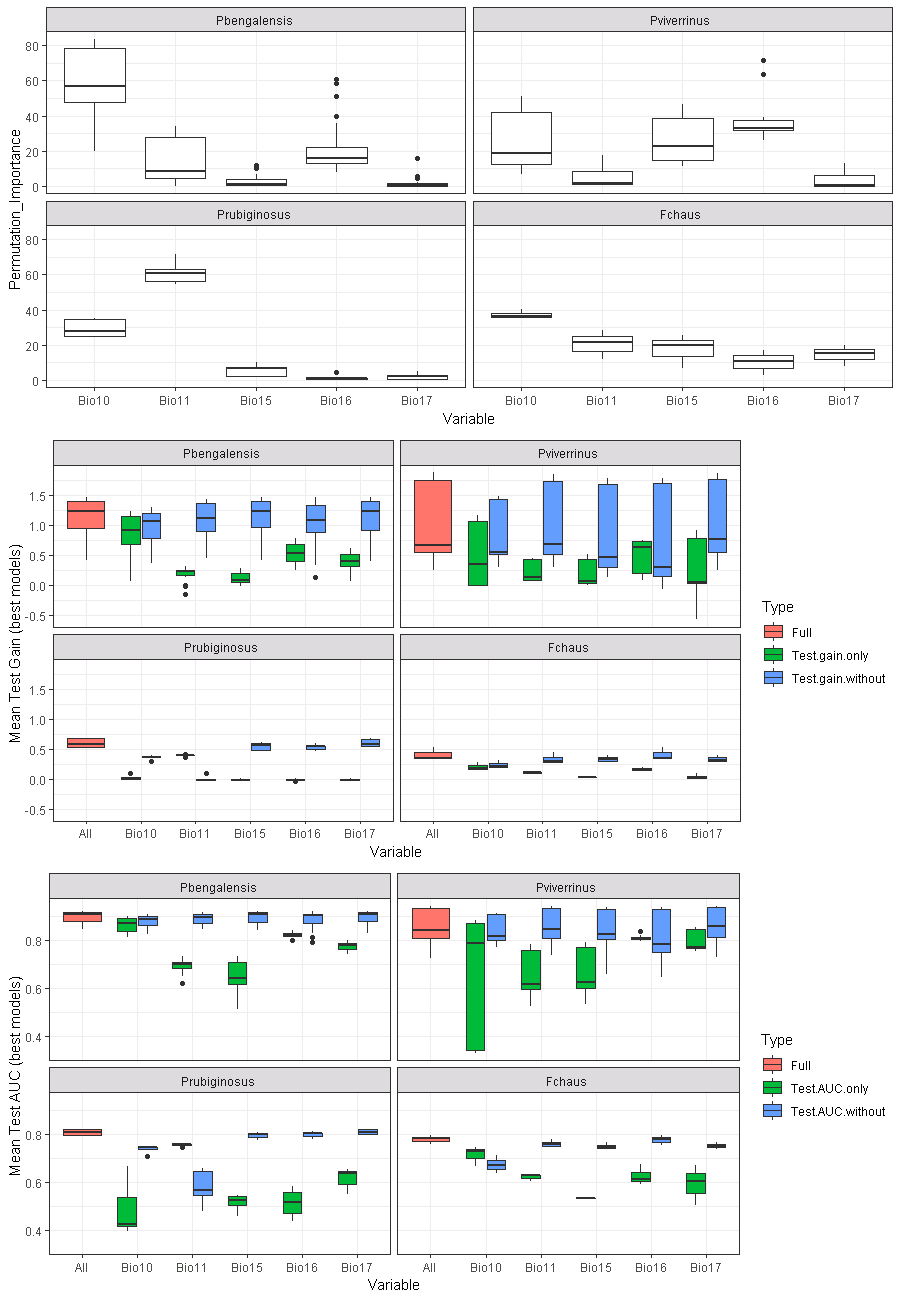
Figure SM1.3** - Variable permutation importance, gain and test AUC values for climate-only models


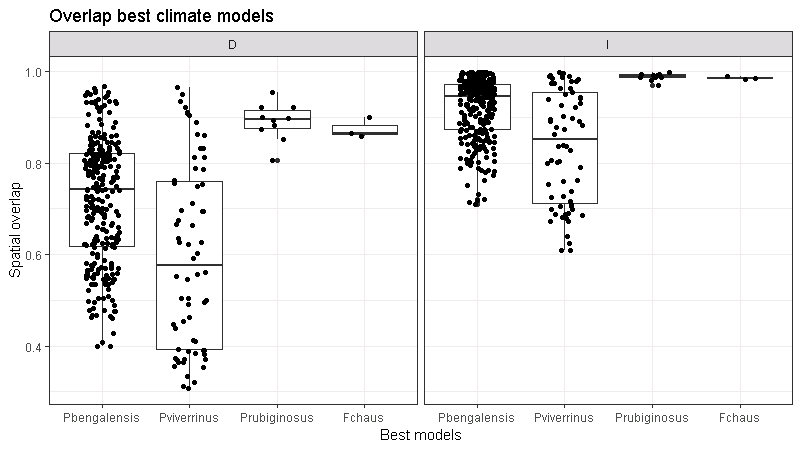


**Figure SM1.4** - Schoener’s overlap metric (D) and the modified Hellinger metric (I) for spatial overlap between best climate-only models.

**Figure SM1.5** - Variable permutation importance, gain and test AUC values for land cover models
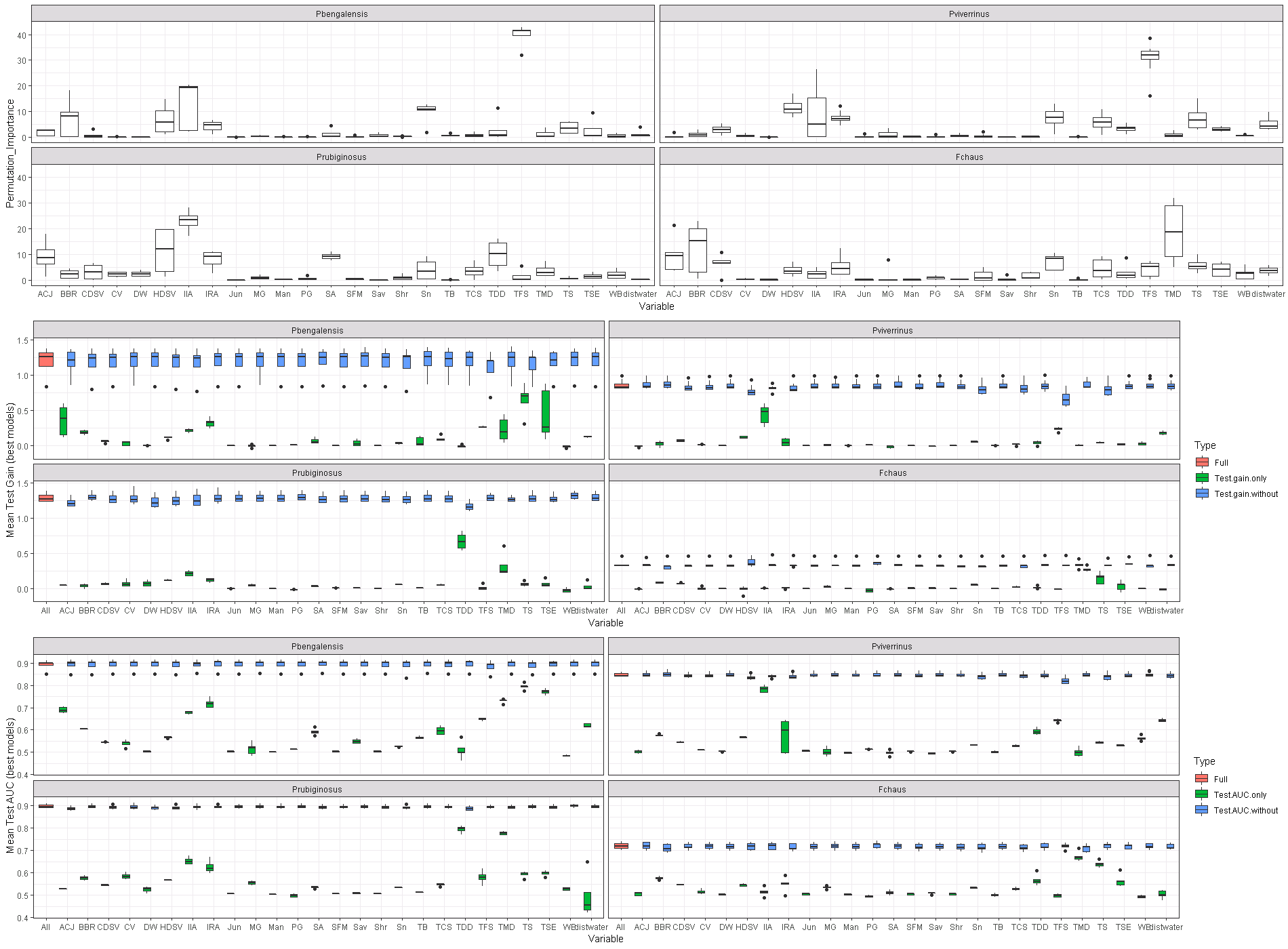


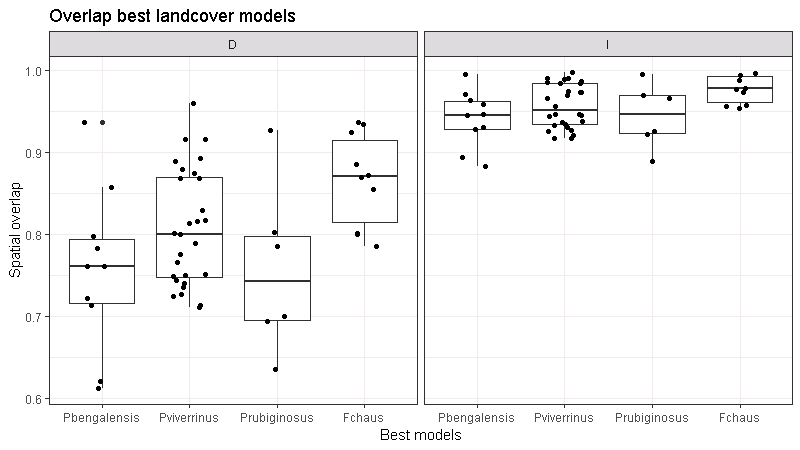


**Figure SM1.6** - Schoener’s overlap metric (D) and the modified Hellinger metric (I) for spatial overlap between best land cover models.

**Figure SM1.7** - Variable permutation importance, gain and test AUC values for human disturbance models
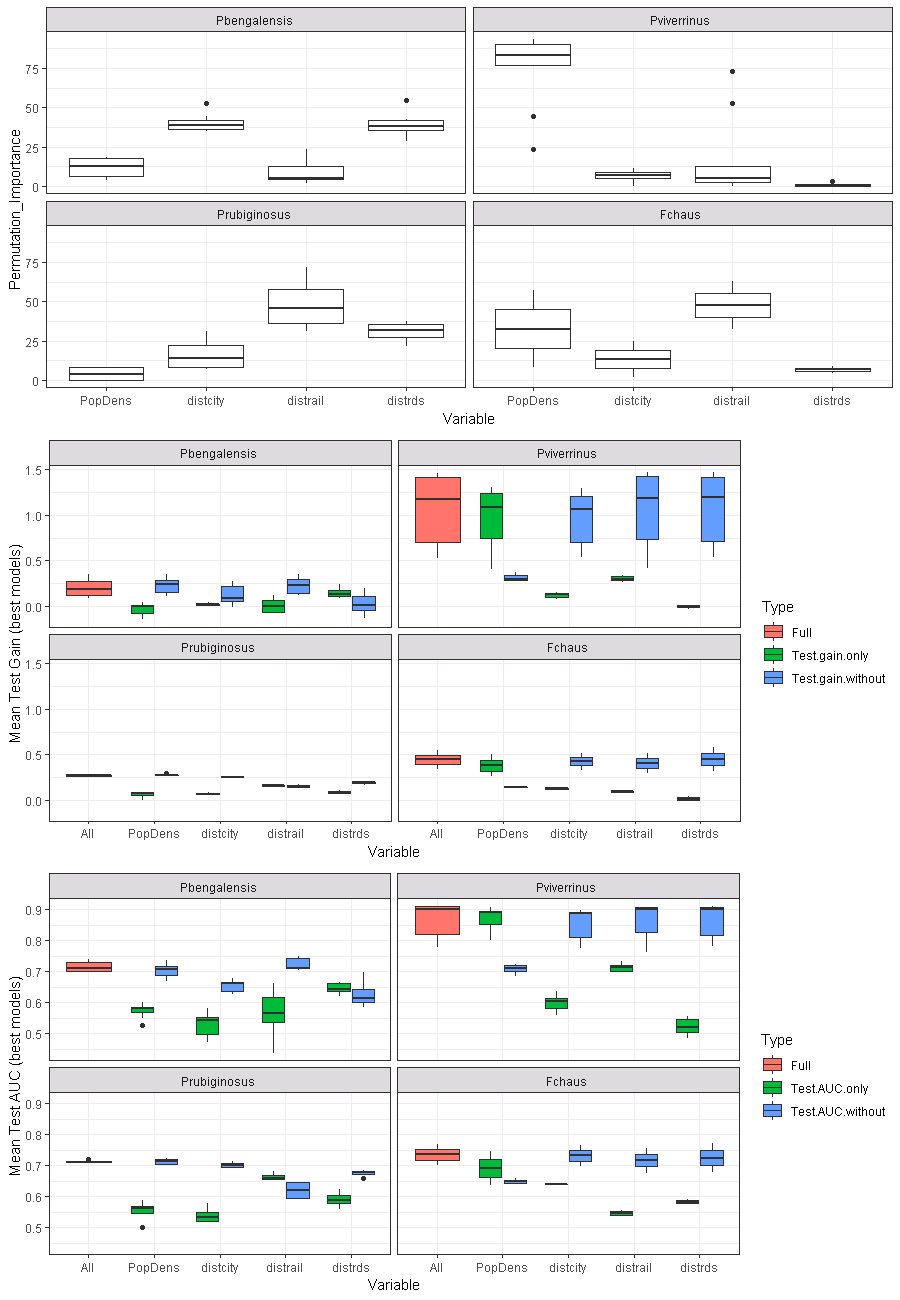


**Figure SM1.8** - Schoener’s overlap metric (D) and the modified Hellinger metric (I) for spatial overlap between best human disturbance models.
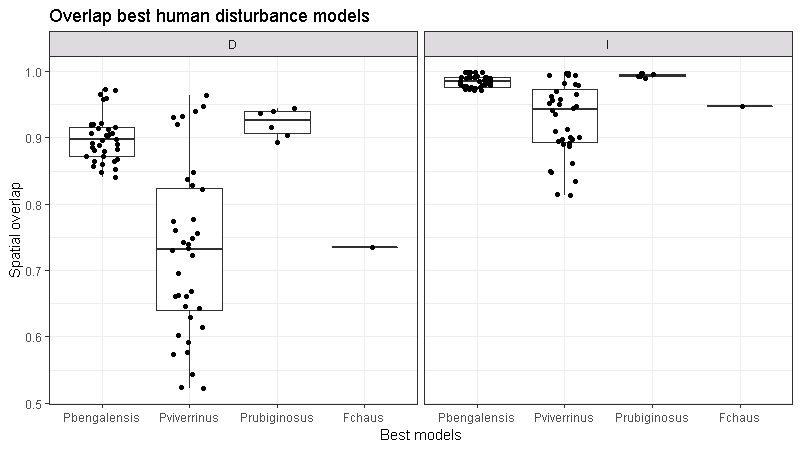


**Figure SM1.9** - Variable permutation importance, gain and test AUC values for prey models
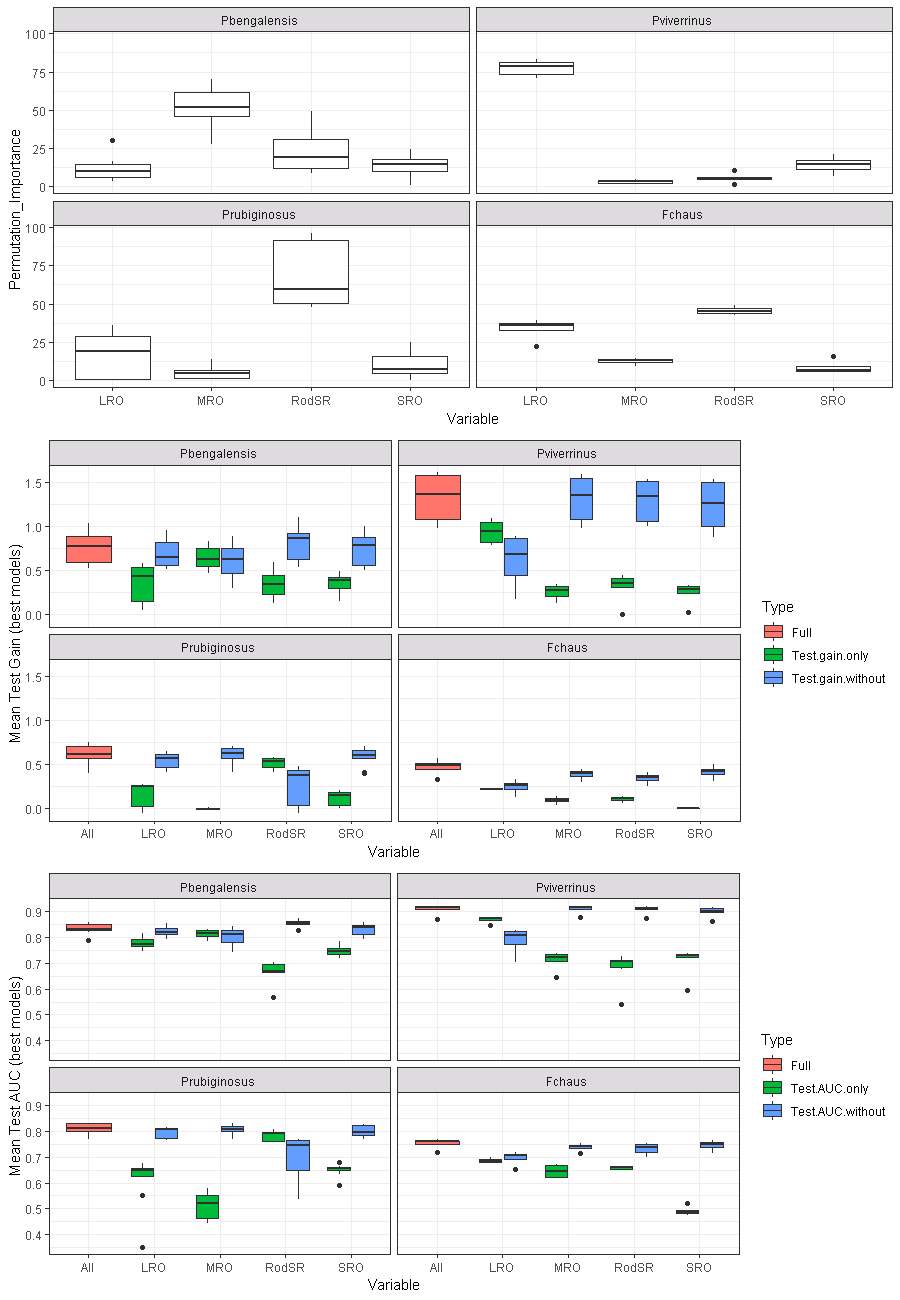


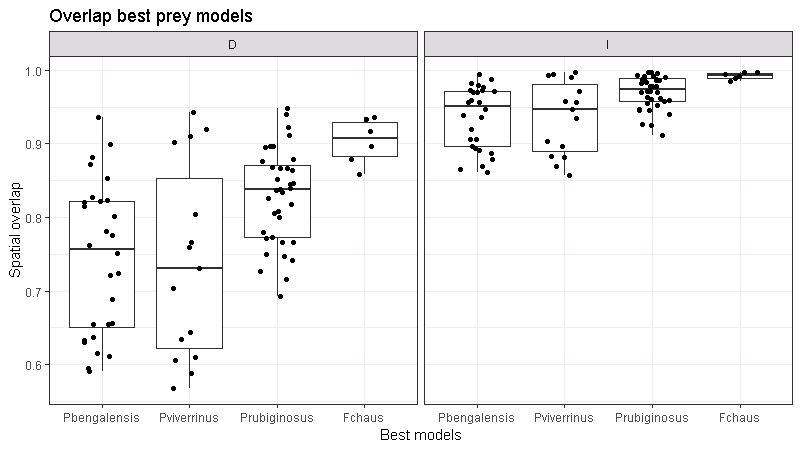


**Figure SM1.10** - Schoener’s overlap metric (D) and the modified Hellinger metric (I) for spatial overlap between best prey models.

**Figure SM1.11** - Regions with mean temperature of warmest quarter (Bio 10) above (red) or below (blue) current time range for predicted past and future climates.
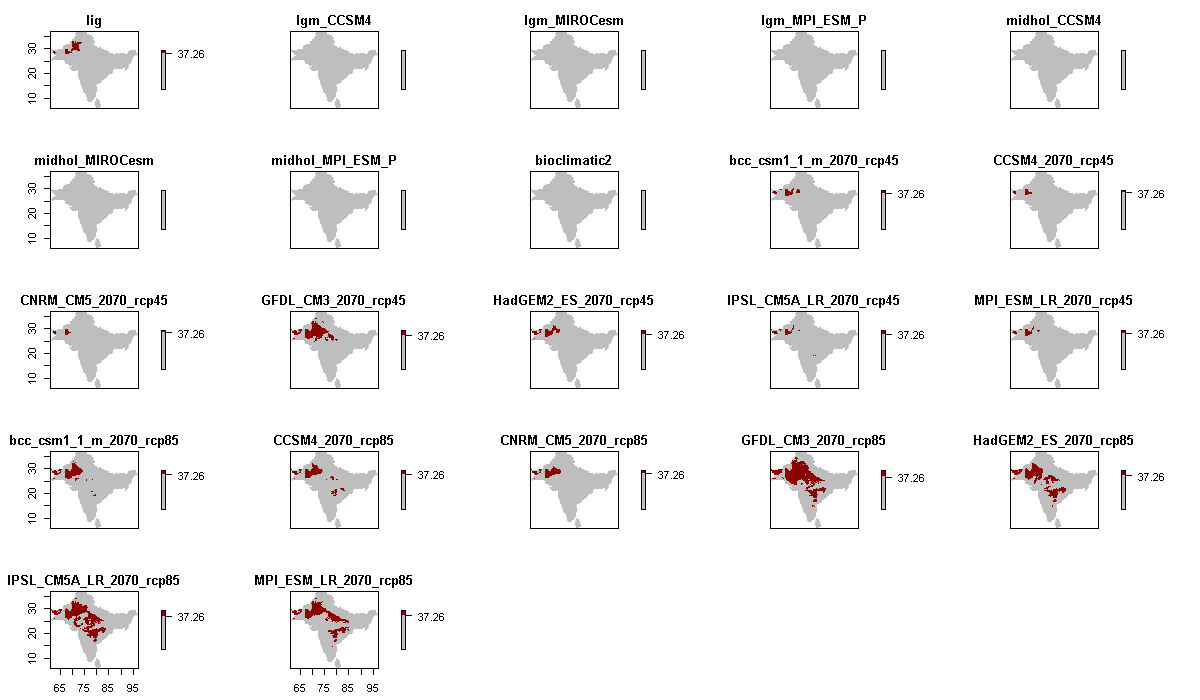


**Figure SM1.12** - Regions with mean temperature of coldest quarter (Bio 11) above (red) or below (blue) current time range for predicted past and future climates.
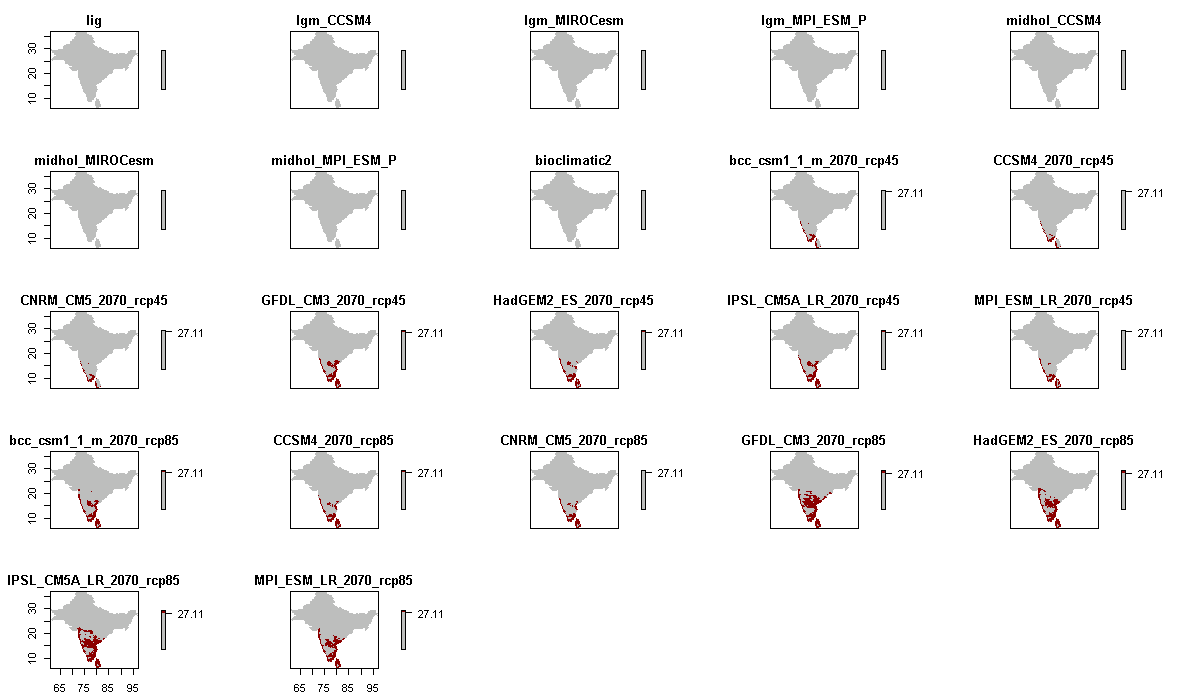


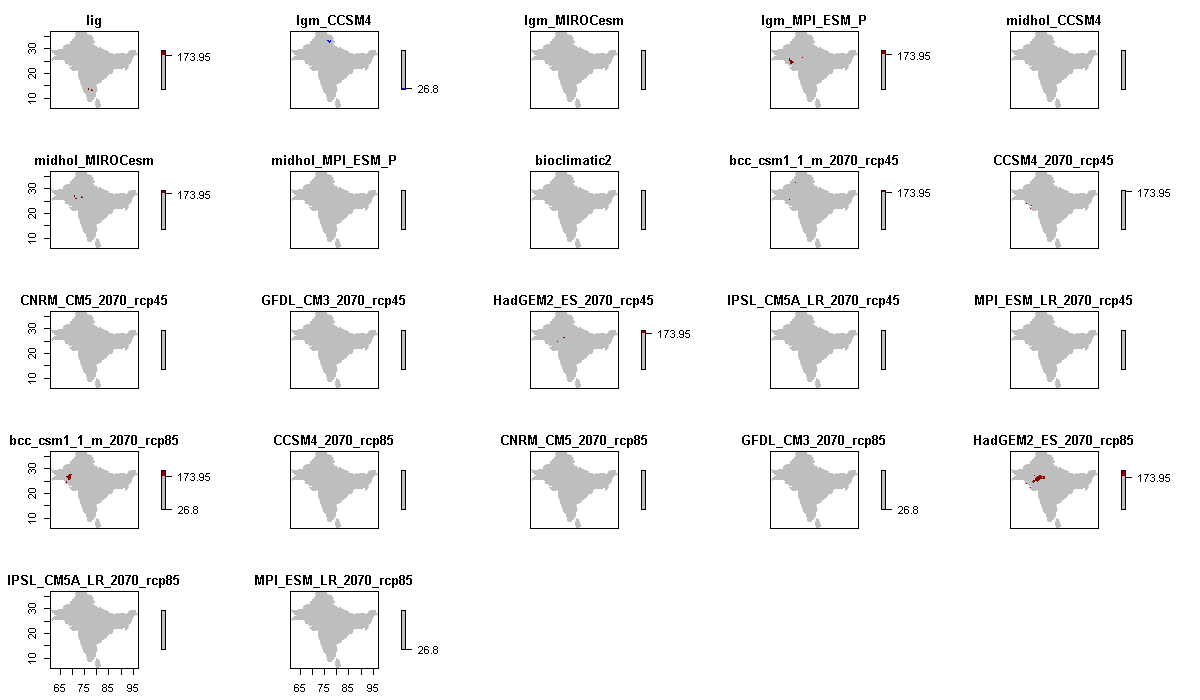


**Figure SM1.13** - Regions with precipitation seasonality (Bio 15) above (red) or below (blue) current time range for predicted past and future climates.

**Figure SM1.14** - Regions with precipitation of wettest quarter (Bio 16) above (red) or below (blue) current time range for predicted past and future climates.
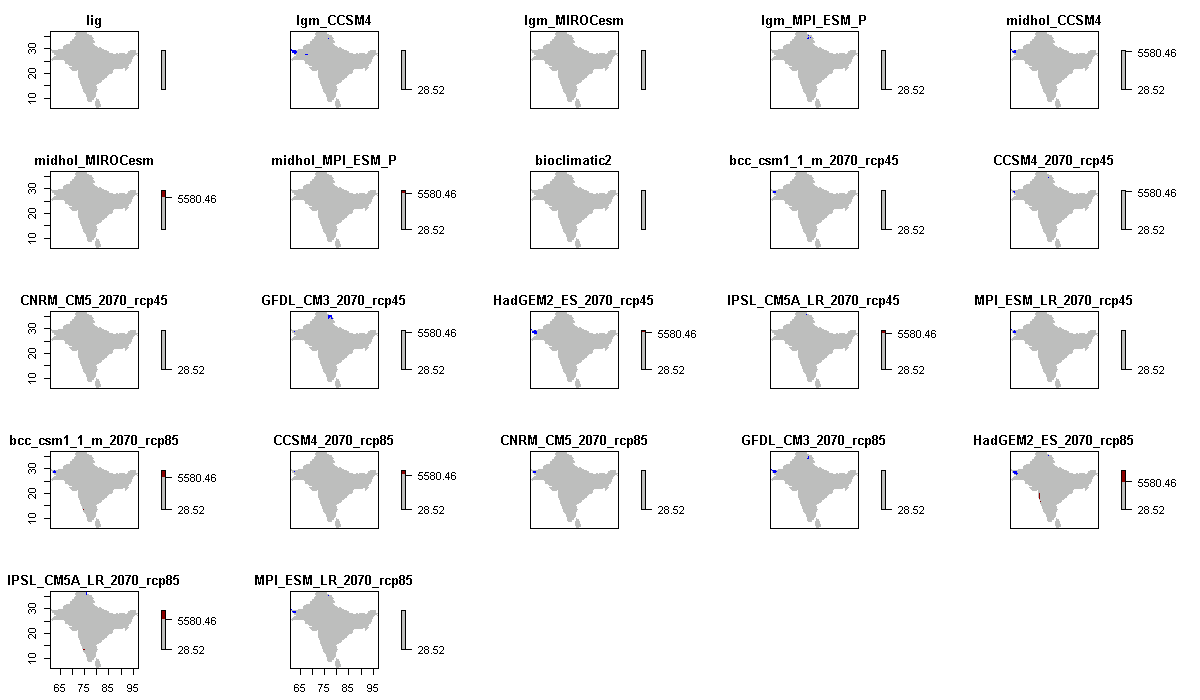


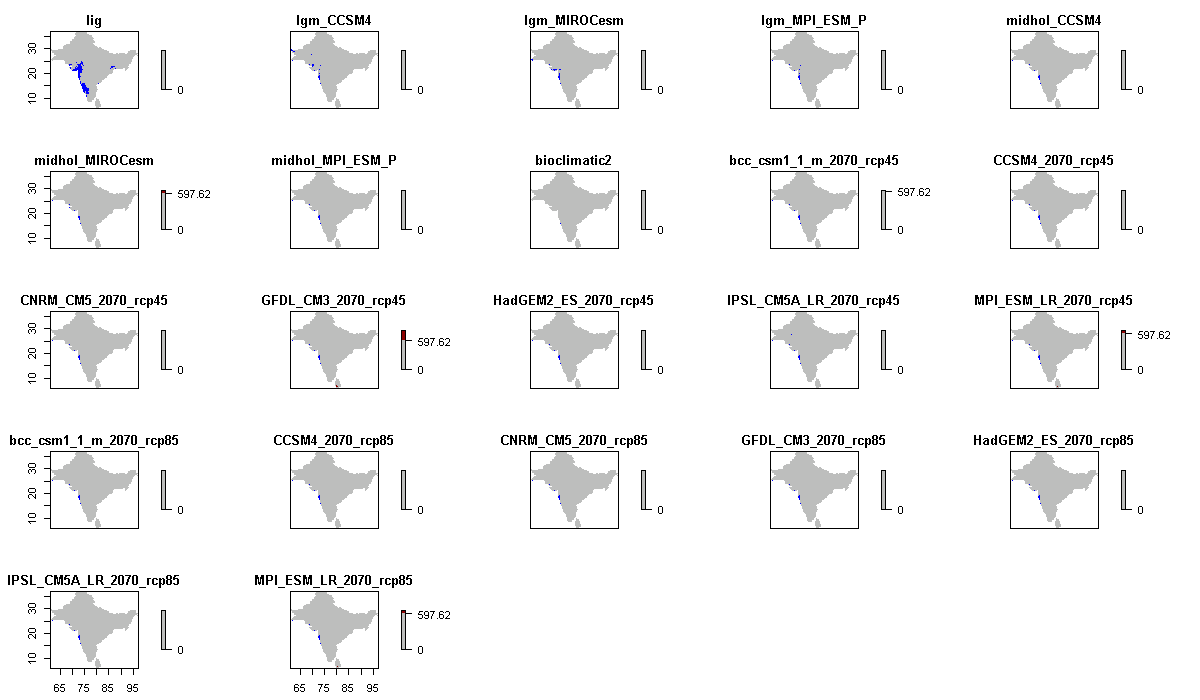


**Figure SM1.15** - Regions with precipitation of driest quarter (Bio 17) above (red) or below (blue) current time range for predicted past and future climates.


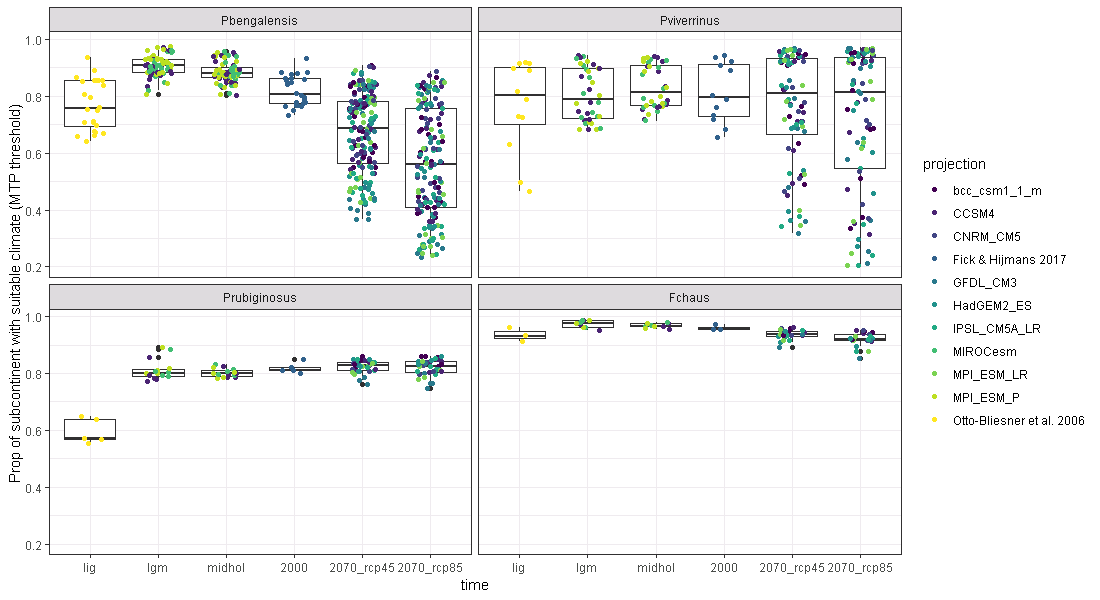


**Figure SM1.16** - Species climatic suitability (MTP threshold) shows species-specific responses within the Indian subcontinent since the last interglacial (LIG; ~120,000 - 140,000 years BP) up to 2070 (based on an optimistic - rcp 45 - and business as usual scenario - rcp 85). Colors display the climate models used for past and future projections. LGM - Last Glacial Maximum (~ 22000 BP); MH - Mid-Holocene (~ 6000 BP); Current time (1950-2000).


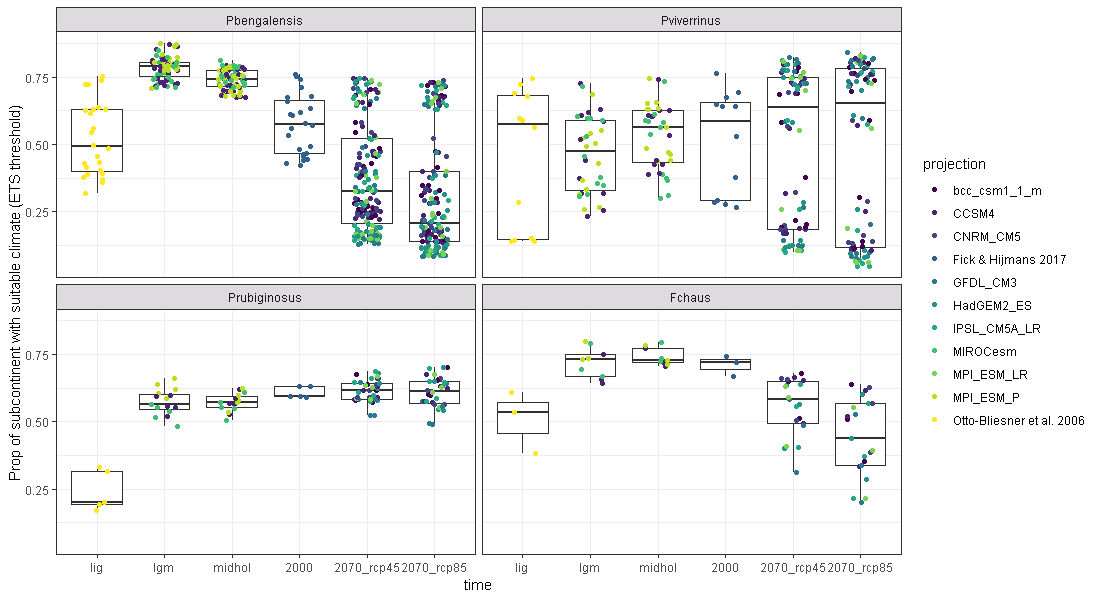


**Figure SM1.17** - Species climatic suitability (ETS threshold) shows species-specific responses within the Indian subcontinent since the last interglacial (LIG; ~120,000 - 140,000 years BP) up to 2070 (based on an optimistic - rcp 45 - and business as usual scenario - rcp 85). Colors display the climate models used for past and future projections. LGM - Last Glacial Maximum (~ 22000 BP); MH - Mid-Holocene (~ 6000 BP); Current time (1950-2000).

**
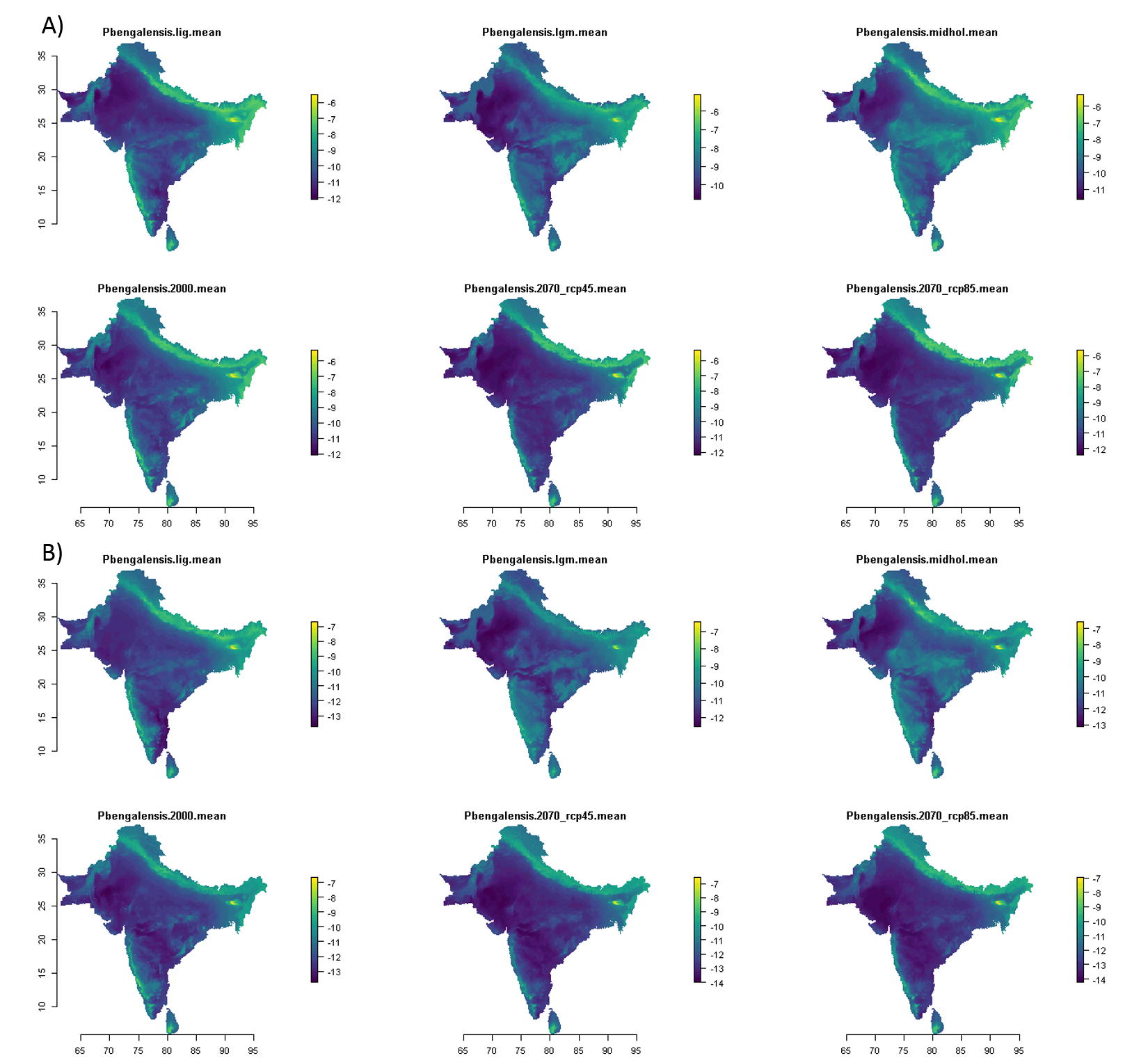
Figure SM1.18** - Mean ensemble for raw (displayed as log(ROR)) predictions (A) and standard deviation (B) for *P. bengalensis*.


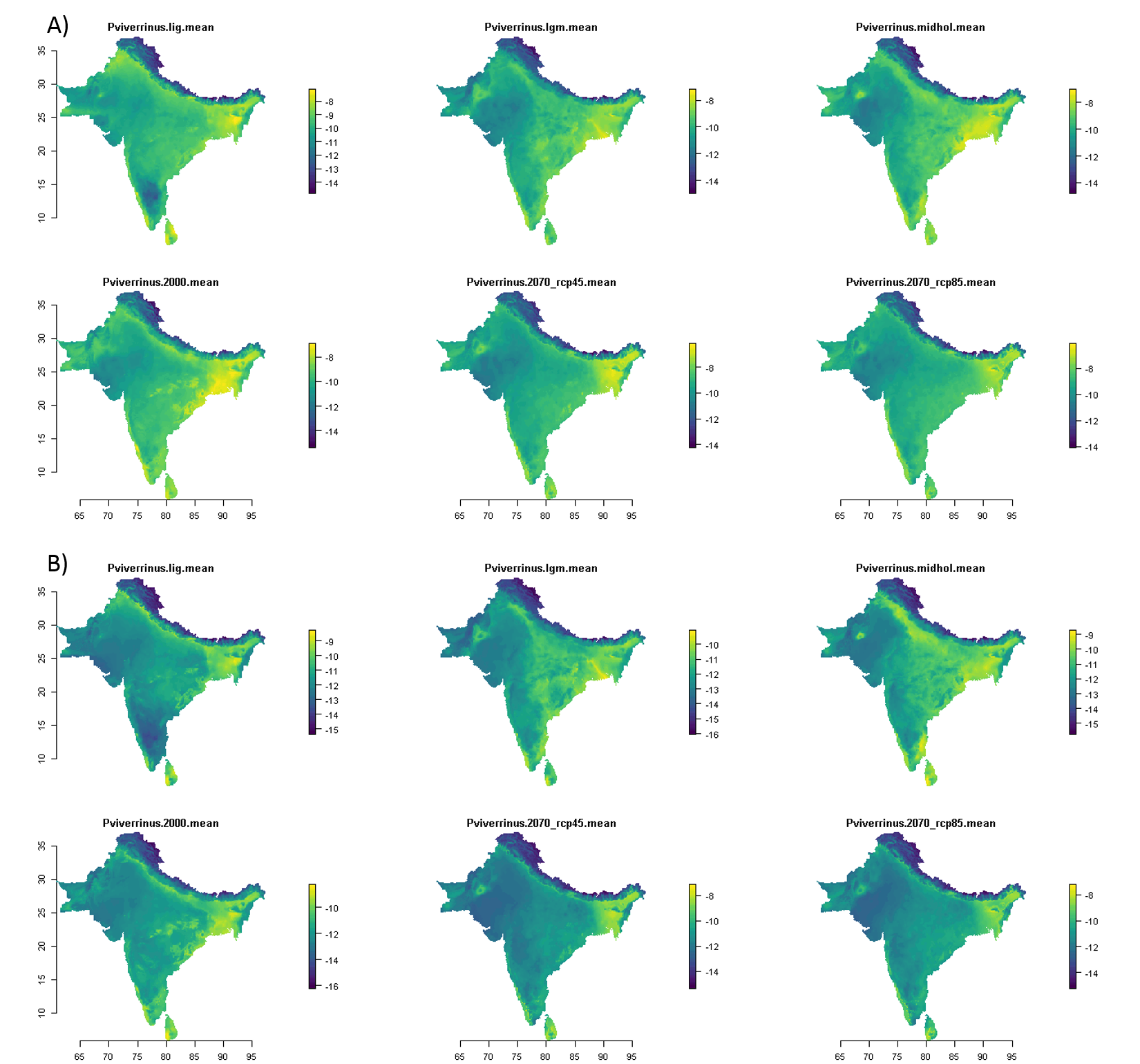


**Figure SM1.19** - Mean ensemble for raw (displayed as log(ROR)) predictions (A) and standard deviation (B) for *P. viverrinus*.

**
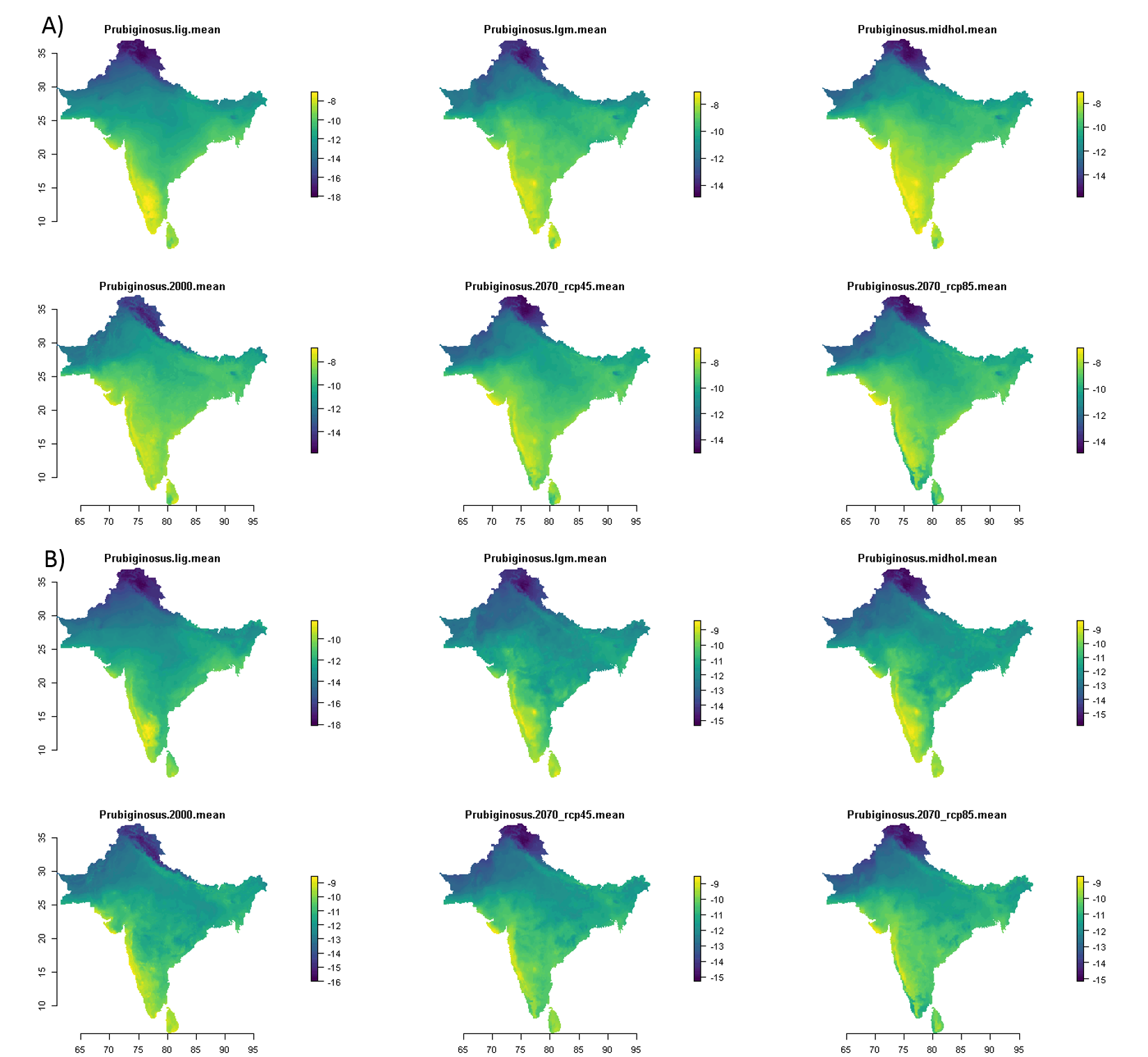
**

**Figure SM1.20** - Mean ensemble for raw (displayed as log(ROR)) predictions (A) and standard deviation (B) for *P. rubiginosus*.


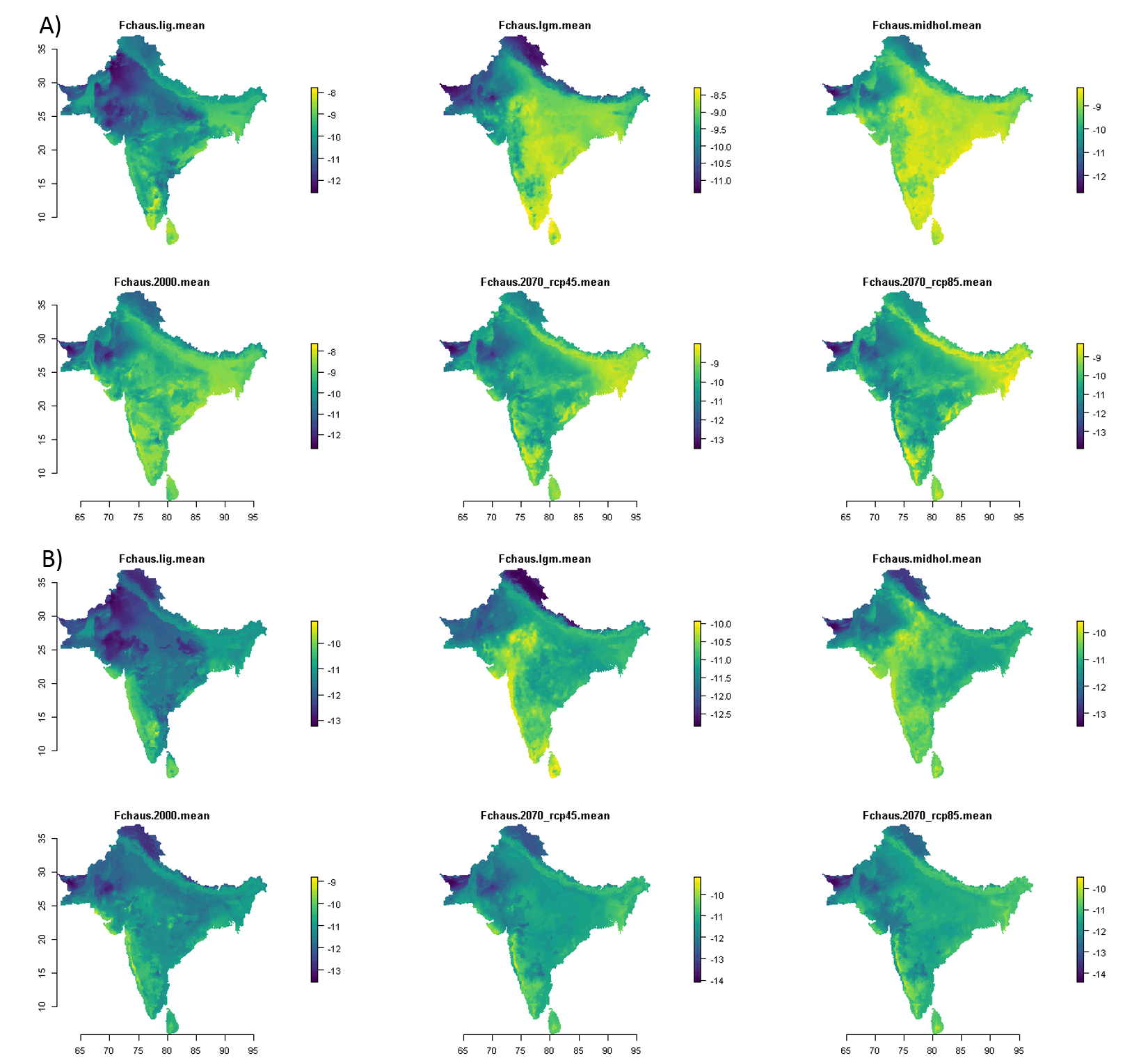


**Figure SM1.21**- Mean ensemble for raw (displayed as log(ROR)) predictions (A) and standard deviation (B) for *F. chaus*.


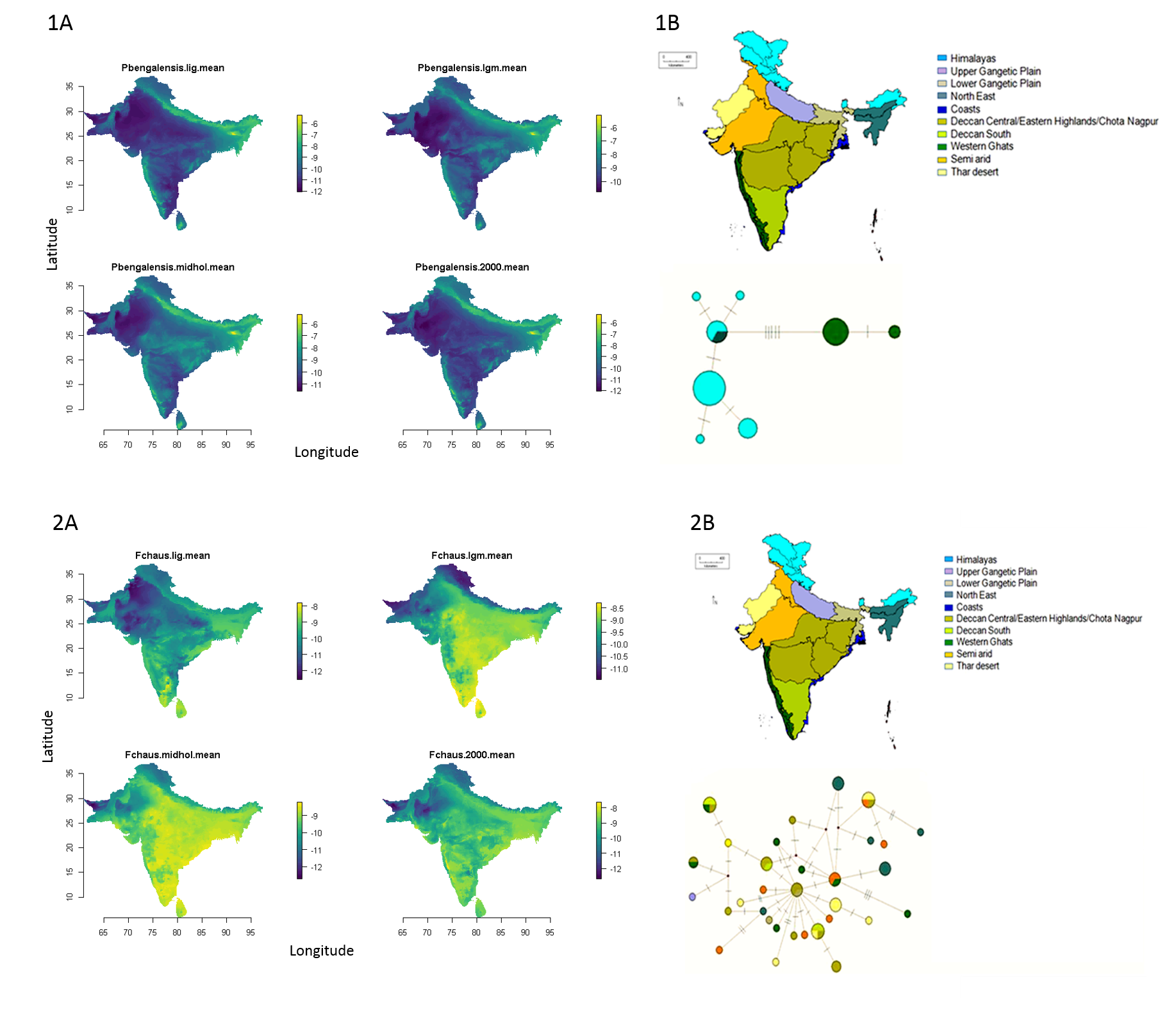


**Figure SM1.22** - Dynamics of past climatic suitability (displayed as log(ROR)) match pre-available information on genetic population structure for *P. bengalensis* (1) and *F. chaus* (2) [^6^](https://paperpile.com/c/fJYCIu/gPilG). Genetic differentiation for the northern and southern populations of *P. bengalensis* in India match with estimated loss of climatic suitability in central India since the LGM (~ 22000 BP) (1B). Time estimates for *F. chaus* population expansion (166K BP - 271K BP) roughly match with predicted increase of climatic suitability within the Indian Subcontinent after the last-interglacial (~120K - 140K years BP) (2B). Median Joining haplotype network for 40 *P. bengalensis* with 362 bp NADH5, 202 bp cytochrome b (1B). Median Joining haplotype network for 55 *F. chaus* with 460 bp NADH5 and 141 bp cytochrome b (2B). Bars on branches denote number of substitutions between connected haplotypes. Size of circle denotes number of individuals in the haplotype. Small circles are missing haplotypes. Adapted from [^6^](https://paperpile.com/c/fJYCIu/gPilG).


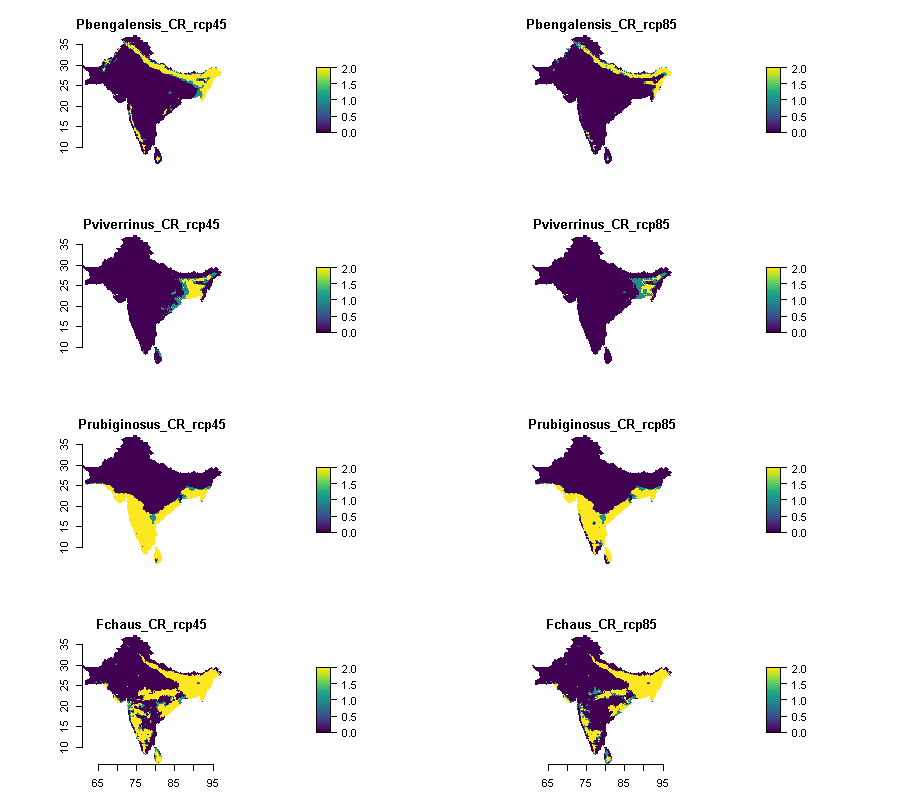


**Figure SM1.23** - Climate refugia, i.e. areas with suitable climate (binary conversion based on P10 threshold) for species occurrence since the Last interglacial (LIG) up to 2070 under two representative conservation pathway scenarios (rcp 45 and rcp 85). Agreement between 75% (blue) and 90% (yellow) of the models is displayed.

**Figure SM1.24** - Test gain for variables included in the best hybrid models.
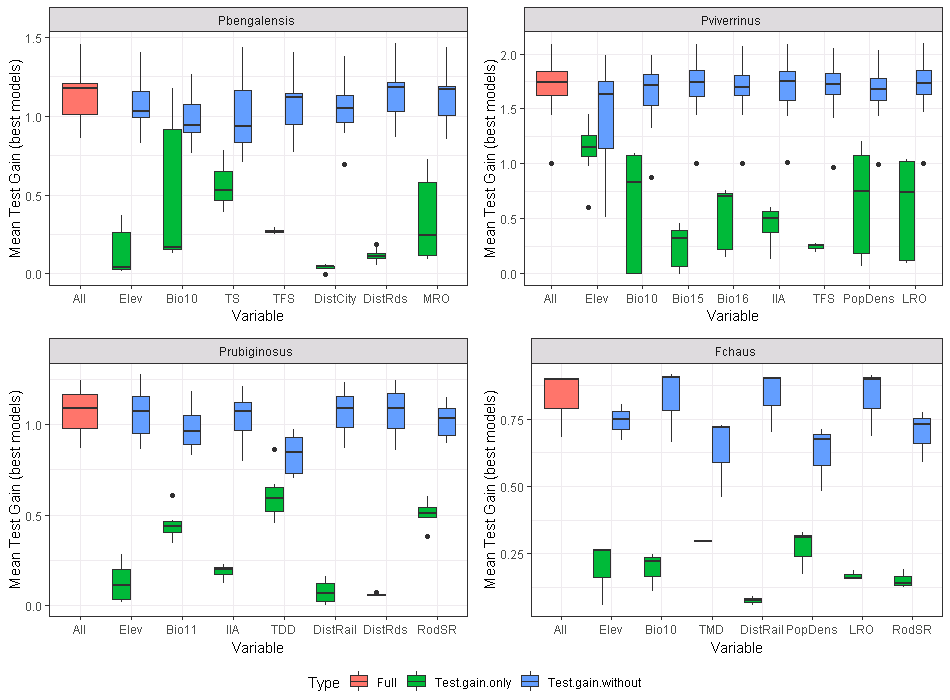


**Figure SM1.25** - Test AUC for variables included in the best hybrid models.
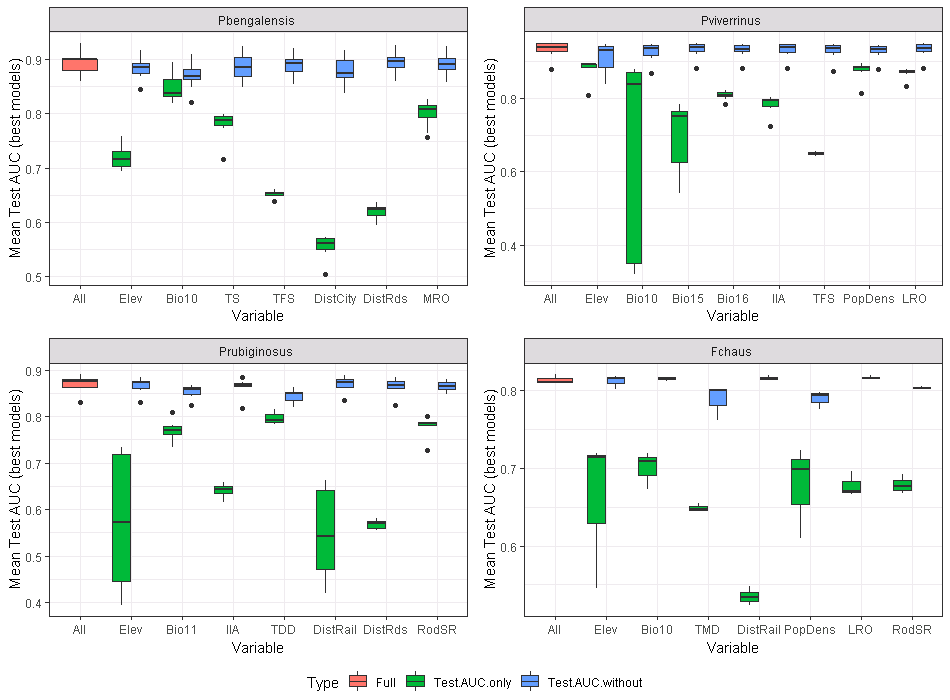


**Figure SM1.26** - Schoener’s overlap metric (D) and the modified Hellinger metric (I) for spatial overlap between best hybrid models.
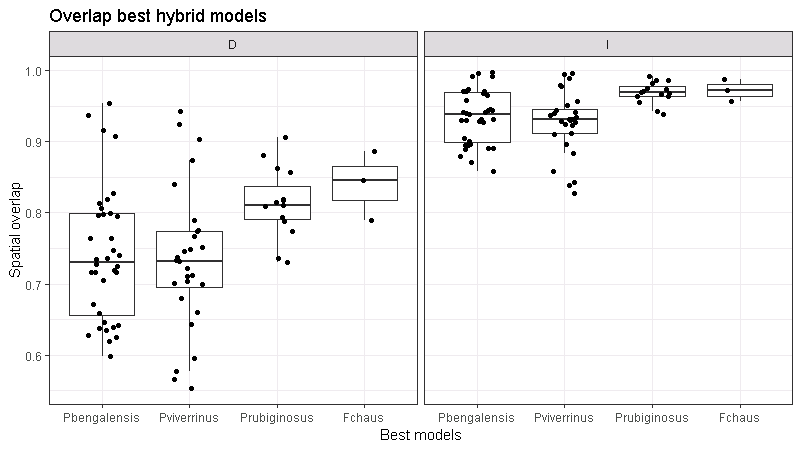

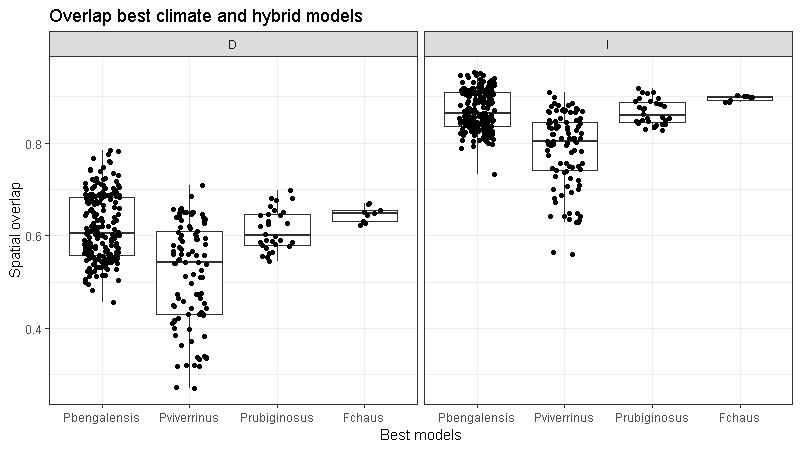


**Figure SM1.27** - Schoener’s overlap metric (D) and the modified Hellinger metric (I) for spatial overlap between best climate-only models and best hybrid models.


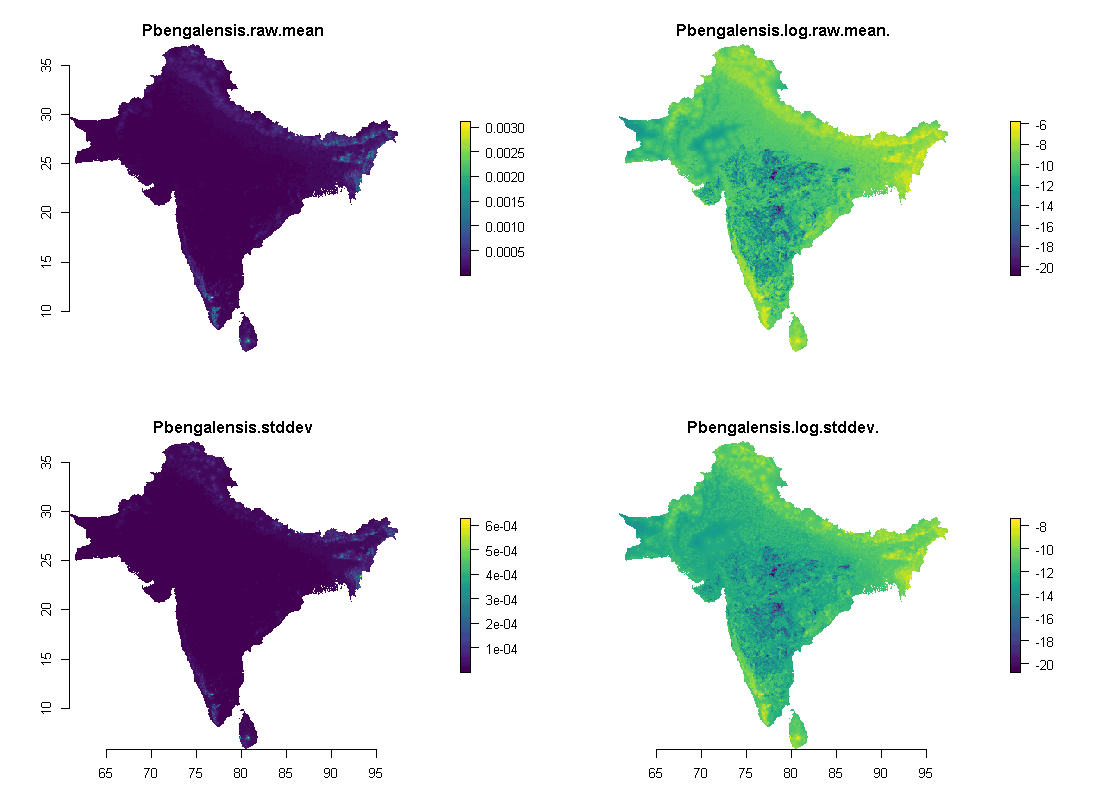


**Figure SM1.28** - *P. bengalensis* raw predictions and standard deviation for best hybrid models together with log transformed rasters for easier interpretation.


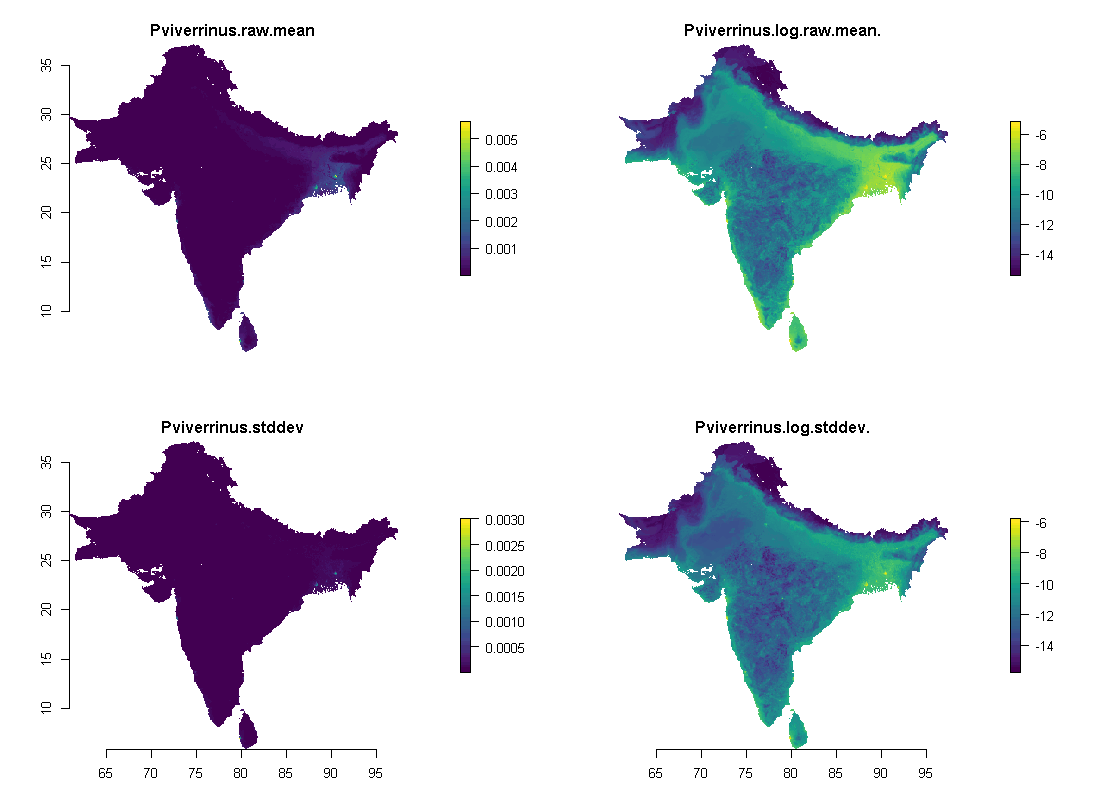


**Figure SM1.29** - *P. viverrinus* raw predictions and standard deviation for best hybrid models together with log transformed rasters for easier interpretation.


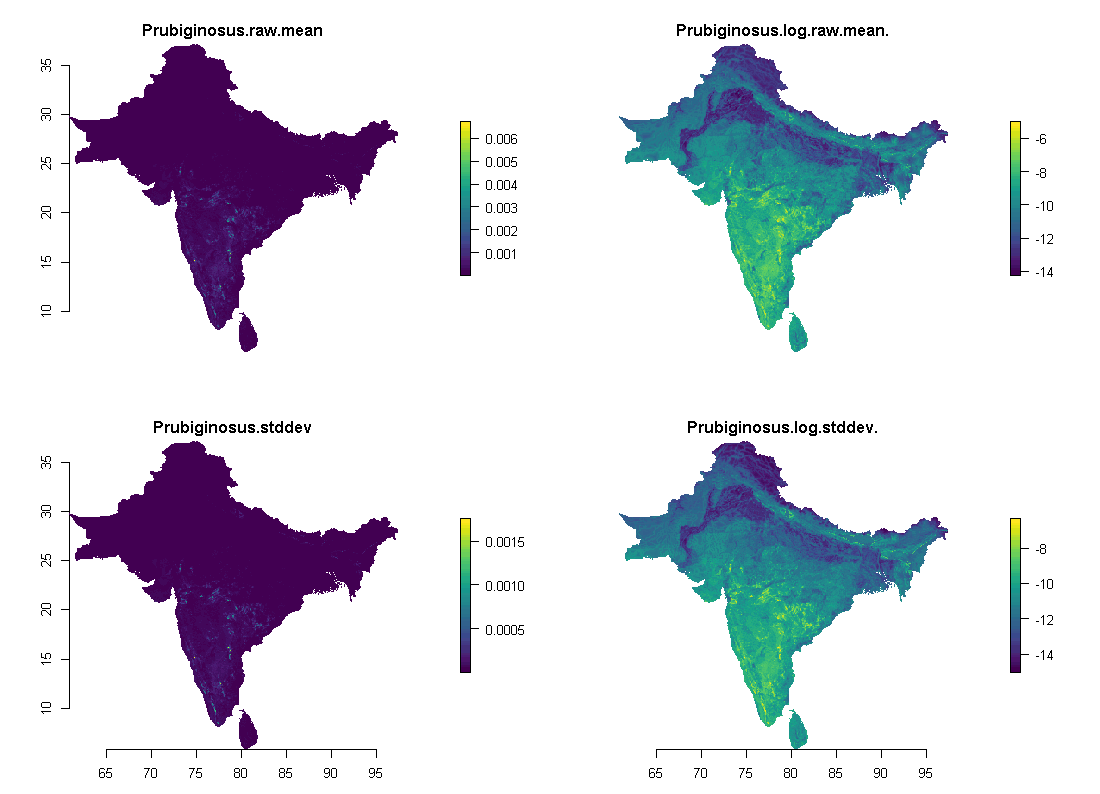


**Figure SM1.30** - *P. rubiginosus* raw predictions and standard deviation for best hybrid models together with log transformed rasters for easier interpretation.


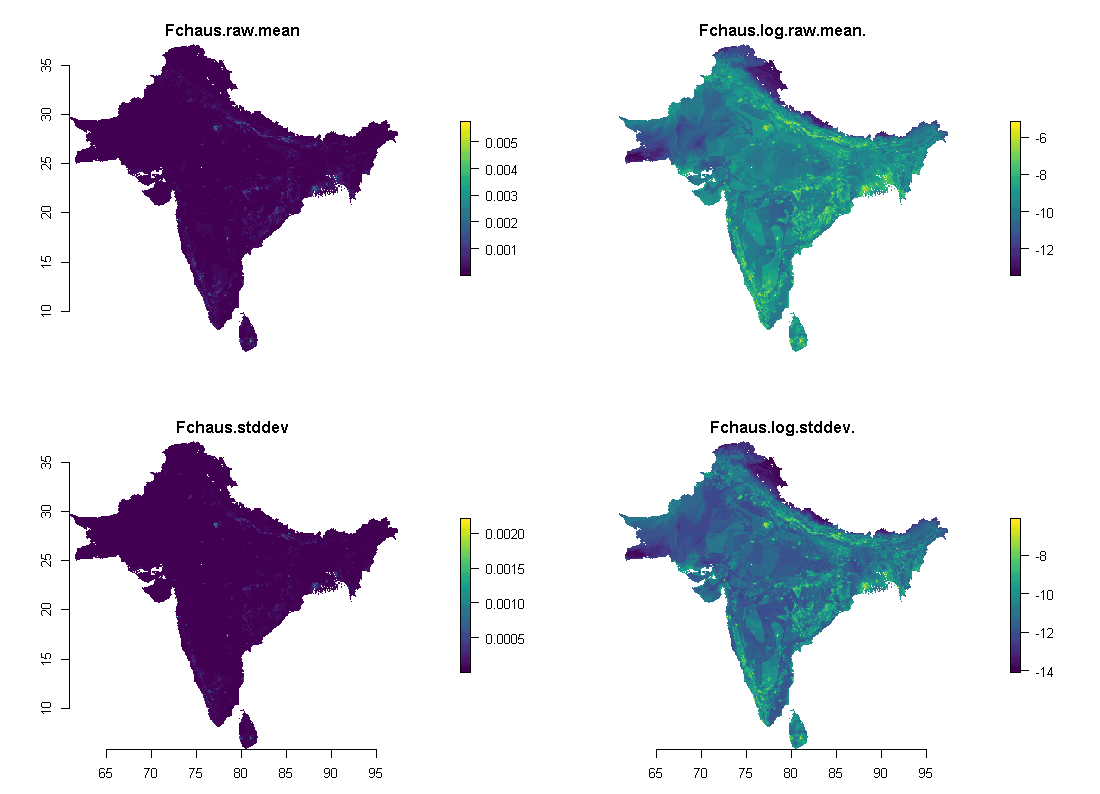


**Figure SM1.31** - *F. chaus* raw predictions and standard deviation for best hybrid models together with log transformed rasters for easier interpretation.


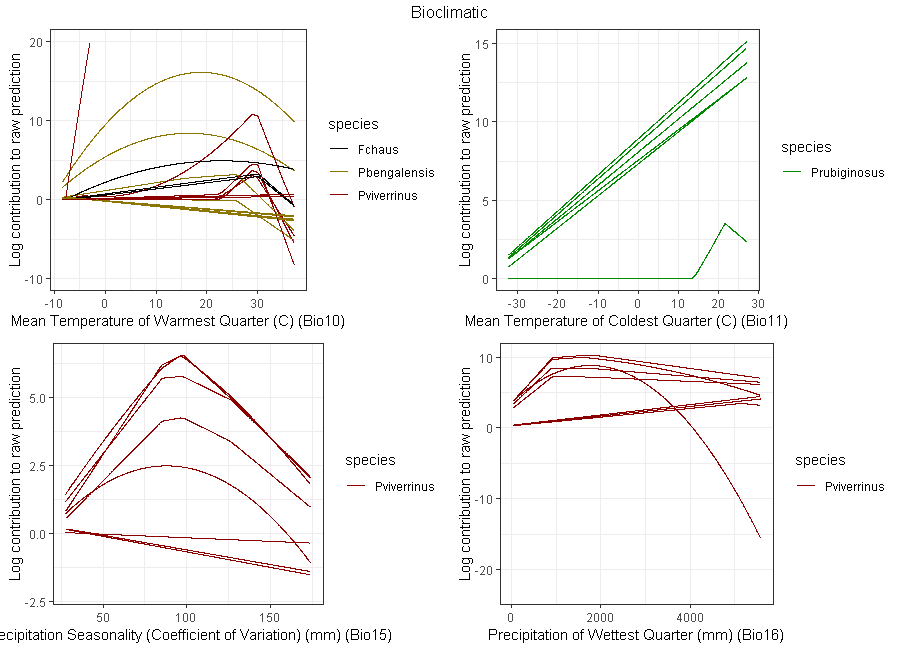


**Figure SM1.32** - Response plots for climatic variables included in the best hybrid models.
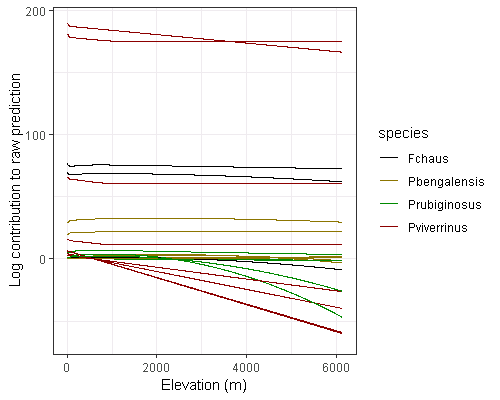


**Figure SM1.33** - Response plots from the best hybrid models for elevation.


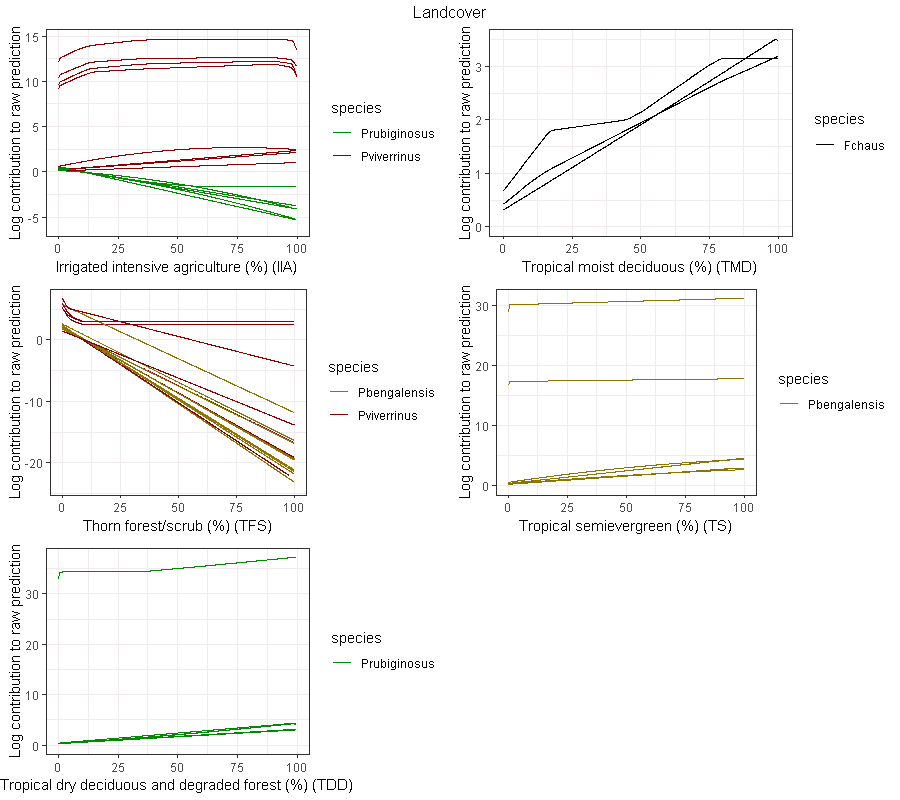


**Figure SM1.34** - Response plots for land cover variables included in the best hybrid models.


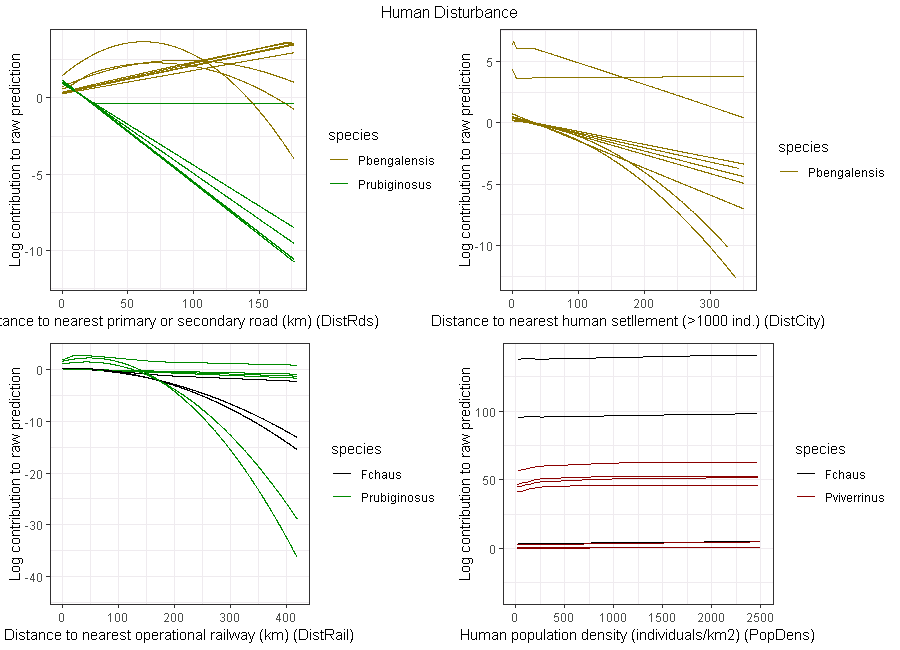


**Figure SM1.35** - Response plots for human disturbance variables included in the best hybrid models.


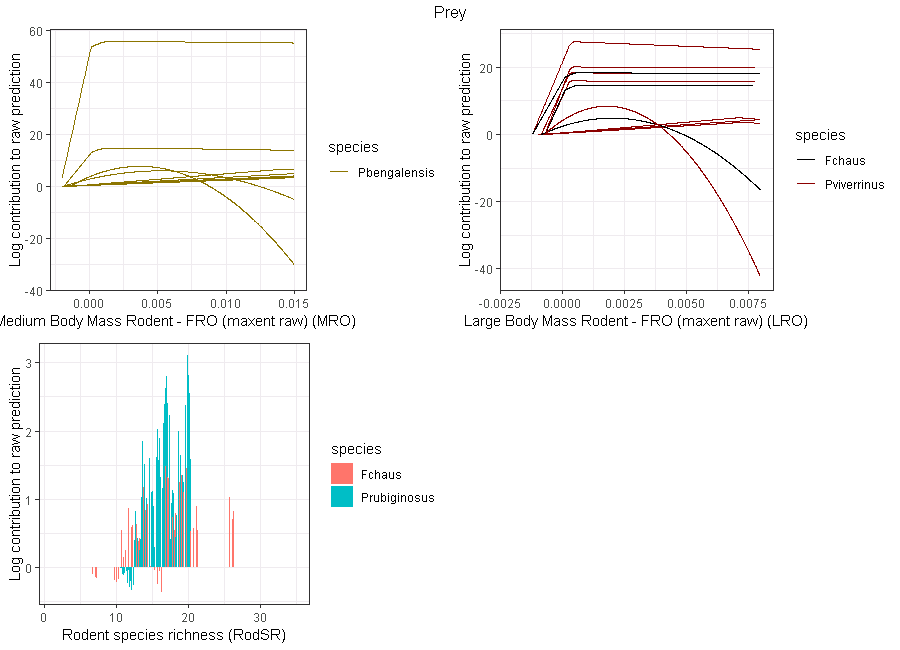


**Figure SM1.36** - Response plots for prey variables included in the best hybrid models.


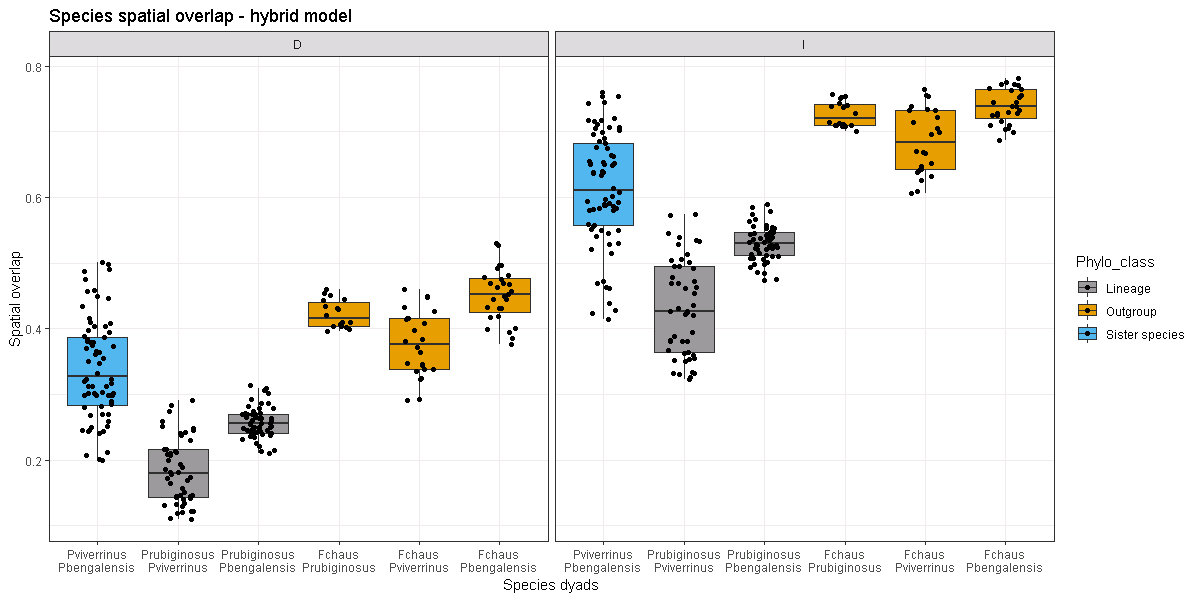


**Figure SM1.37** - Schoener’s overlap metric (D) and the modified Hellinger metric (I) for spatial overlap between species using the best hybrid models.


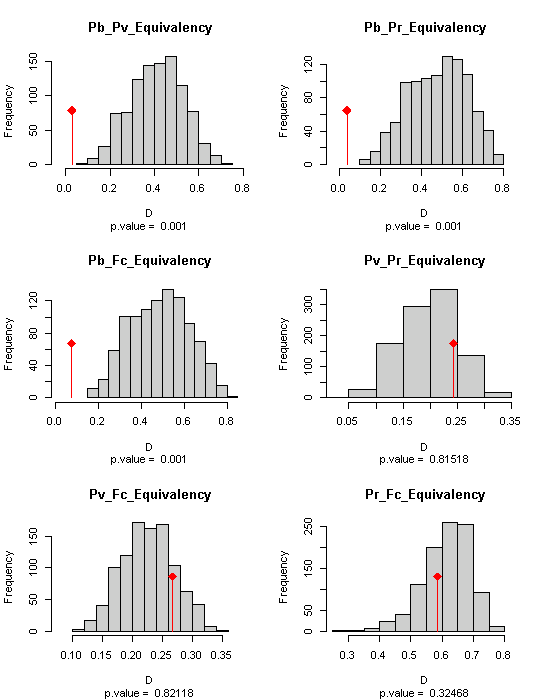


**Figure SM1.38** - Niche equivalency test comparing observed Schoener’s overlap metric (red line) and simulated values within full (hybrid) environmental space.


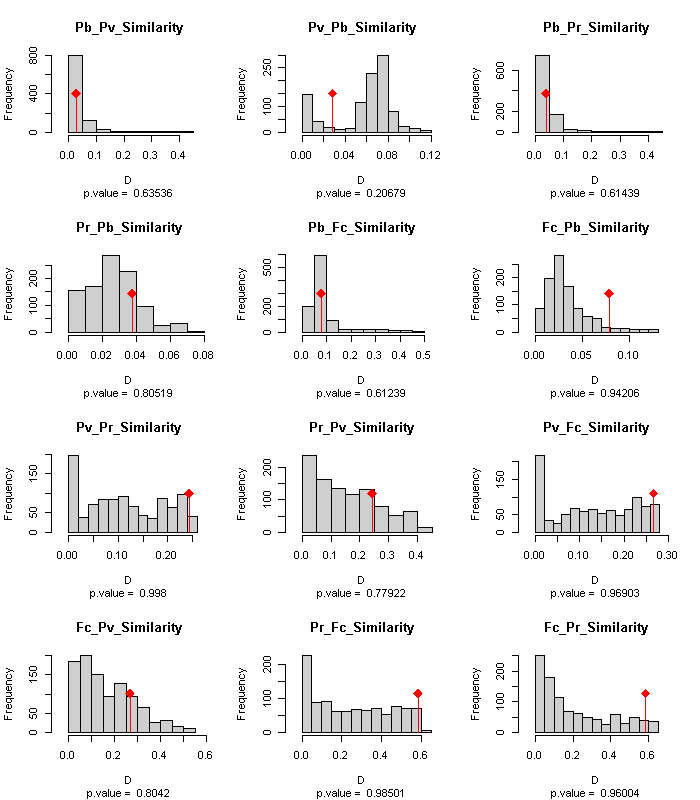


**Figure SM1.39** - Niche similarity test comparing observed Schoener’s overlap metric (red line) and simulated values within full (hybrid) environmental space.

**
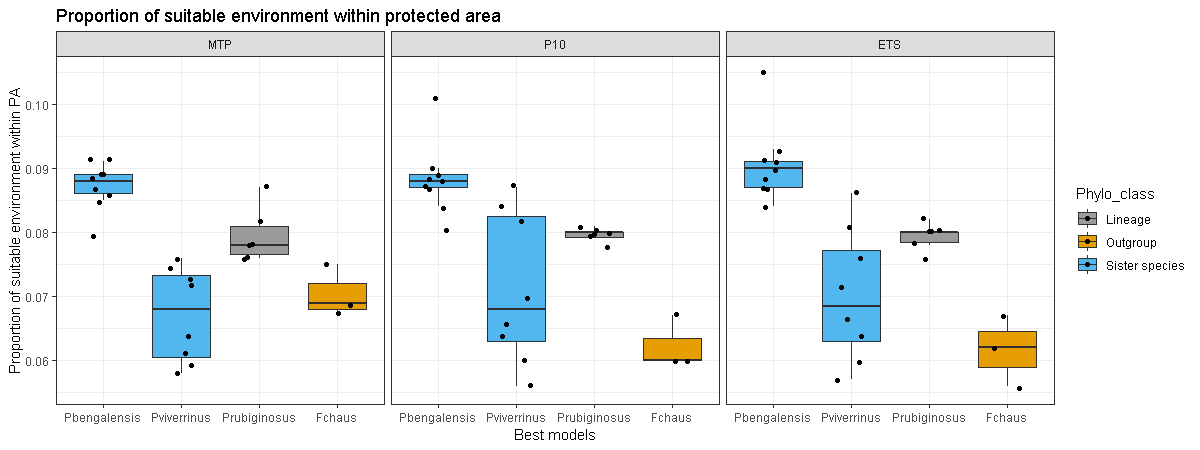
**

**Figure SM1.40** - Proportion of suitable environment predicted by the best hybrid models covered by protected areas. Overall pattern is similar among the different thresholds (MTP, P10, ETSS) used.

**References**

1. [Galante, P. J. *et al.* The challenge of modeling niches and distributions for data-poor species: a comprehensive approach to model complexity. *Ecography*  **41**, 726–736 (2017).](http://paperpile.com/b/fJYCIu/KRsQo)

2. [Elith, J. *et al.* A statistical explanation of MaxEnt for ecologists. *Diversity and Distributions* **17**, 43–57 (2010).](http://paperpile.com/b/fJYCIu/kYQ0I)

3. [Merow, C., Smith, M. J. & Silander, J. A. A practical guide to MaxEnt for modeling species’ distributions: what it does, and why inputs and settings matter. *Ecography*  **36**, 1058–1069 (2013).](http://paperpile.com/b/fJYCIu/9zWOi)

4. [Radosavljevic, A. & Anderson, R. P. Making better Maxentmodels of species distributions: complexity, overfitting and evaluation. *J. Biogeogr.* **41**, 629–643 (2013).](http://paperpile.com/b/fJYCIu/qtqec)

5. [Phillips, S. *A brief tutorial on Maxent*.](http://paperpile.com/b/fJYCIu/MlgLm) <http://www.cs.princeton.edu/~schapire/maxent/tutorial/tutorial.doc> [(2006).](http://paperpile.com/b/fJYCIu/MlgLm)

6. [Mukherjee, S. *et al.* Ecology driving genetic variation: a comparative phylogeography of jungle cat (Felis chaus) and leopard cat (Prionailurus bengalensis) in India. *PLoS One* **5**, e13724 (2010).](http://paperpile.com/b/fJYCIu/gPilG)
