# Supplementary material for: Closely related species show species-specific environmental responses and different spatial conservation needs: Prionailurus cats in the Indian subcontinent
Source: Sci Rep. 2020 Oct 30;10:18705. doi: 10.1038/s41598-020-74684-8 (PMC7599212; doi:10.1038/s41598-020-74684-8)
Supplement: Supplementary file 3 — Supplementary Information 2. [file 41598_2020_74684_MOESM3_ESM.docx]

**SM2 - supplementary material for**

**Title**: Closely related species show species-specific environmental responses and different spatial conservation needs: *Prionailurus* cats in the Indian subcontinent

**Authors**: André P. Silva, Shomita Mukherjee, Uma Ramakrishnan, Carlos Fernandes, Mats Björklund

**Modelling rodent occurrence across the Indian Subcontinent**

**Methods**

**Target species and occurrence data**

To identify rodent species preyed by small and medium sized carnivores we consulted trophic ecology literature from South / South-East Asia and South of China (Supplementary table SM2.1). Prey identification at the species level was rare and/or geographically limited in most studies, therefore records were pooled by genus. We identified the following genera preyed by small carnivores: *Apodemus*, *Bandicota*, *Cannomys*, *Eothenomys*, *Golunda*, *Hyperacrius*, *Leopoldamys*, *Maxomys*, *Meriones*, *Mus*, *Nesokia*, *Niviventer*, *Rattus*, *Rhizomys*, *Tatera.*

We excluded members of the Hystricidae, Sciuridae and Gliridae families since there is no evidence that these contribute to the bulk of the small wild cat diet (our main focus). In addition, we excluded genera not present or with very restricted distribution within the Indian subcontinent (*Maxomys*, *Eothenomys* and *Hyperacrius*). For the remaining genera, we extracted occurrence records, including only those with information on collection date and geographic location, from the Global Biodiversity Information Facility (http://www.gbif.pt/), published literature [^1^](https://paperpile.com/c/87qg9w/GmW9v) and personal communications (Krishnapriya Tamma, unpublished results; Amruta Varudkar, unpublished results, Sahila Kudalkar, unpublished results). We found records available for all genera except for the *Rhizomys* genus. The genus *Leopoldamys* was discarded due to low number of occurrence records (n = 5). To represent possible different energetic intakes for small wild cats, records were then classified into three body size classes (small sized rodents ≤ 70 g; medium sized rodents between 70 g and 150 g; large sized rodents > 150 g) (Supplementary Table SM2.2, Supplementary Fig. SM2.1). Species’ adult body mass was collected from PanTHERIA [^2^](https://paperpile.com/c/87qg9w/zRfaD) and other published literature [^3^](https://paperpile.com/c/87qg9w/HesB9). When information for body mass was available in both sources and they differed, we calculated the mean value. Rodent occurrence records used in the following modelling procedures are deposited in Dryad (DOI: XXX)

**Environmental predictors**

The ecological niche of the selected rodent genera was modeled based on three environmental components. Topographic (elevation) and bioclimatic variables from the WorldClim data set (Bio10, Bio11, Bio15, Bio16, Bio17), land cover categories from the Global Land Cover Map 2000 (including human settlements) and human population estimates (PopDens) (see Supplementary Table SM1.1 for details on environmental variables). Only moderately correlated (r < 0.55) environmental variables were included in the models. Between correlated variables, the ones that represent extreme environmental variations and are of easier biological interpretation were retained. We also gave preference to annual and quarterly bioclimatic variables since these are more transferable to future climatic predictions (see MaxEnt Tutorial), also facilitating the use of these models in future studies. All variables were resampled to a 10 km resolution to match the accuracy of the species occurrence data and the resolution used to model the small cats’ ecological niche.

**Filtering strategies and bias files**

Data clustering and bias can influence environmental niche model predictions, and therefore data spatial filtering has been recommended as an effective measure to decrease potential bias [^4,5^](https://paperpile.com/c/87qg9w/hbHLw+K6FJW) . We first plotted occurrence records on the Indian subcontinent map and removed erroneous or inaccurate geo-referenced locations. In addition, all records prior to 1970 were discarded in an attempt to decrease the mismatch between the occurrence records and the environmental data (from the late 90/2000s). Following recommendations [^6^](https://paperpile.com/c/87qg9w/fwpra), we prepared two differently filtered data sets (Supplementary Table SM2.3). In the first one (RD) all duplicated records within each cell (10 x 10 km) were removed (Supplementary Fig. SM2.2). A second data set (RDbal) was built to have similar point density across the study area (balanced design) (Supplementary Fig. SM2.2). For every administrative area (Bangladesh, Bhutan, Nepal, Pakistan, Sri Lanka, and Indian administrative states) we calculated the average record density for each genus (excluding administrative areas without detections). For administrative areas with more occurrence records than the average we randomly removed records until achieving similar density between administrative areas.

MaxEnt assumes that species occurrence data are unbiased, independent samples from the distribution of the species (see MaxEnt tutorial). However, occurrence records are more likely to be collected closer to human settlements and in areas of easier access (e.g. closer to roads and agricultural fields). To correct for possible bias in our data sets, we further produced two different types of bias files (BM01 and BM001) (following the same approach [^6^](https://paperpile.com/c/87qg9w/fwpra), for BM01, non-sampled 10 x 10 km cells were given a value of 0.1, while for BM001 the same cells received a value of 0.01. In both files a value of 1 was given for cells with occurrence records (i.e. sampled cells).

**MaxEnt modeling**

Rodent occurrence was modelled using a maximum entropy based machine learning algorithm that estimates the probability distribution for a species occurrence based on environmental constraints [^7^](https://paperpile.com/c/87qg9w/J1wAJ). This algorithm was selected since it has been demonstrated to be one of the most robust when using only presence records [^8^](https://paperpile.com/c/87qg9w/OtrCa). Models were built using previsouly suggested parameters [^7^](https://paperpile.com/c/87qg9w/J1wAJ), namely auto feature selection, a random seed (i.e. MaxEnt will generate a new set of random test points for each replicate), 10,000 background points, and calibration using 70% of the available records with the remaining 30% used for model evaluation. The partitioning of the calibration and evaluation data set was random. We ran 10 replicates with 5000 iterations for each model and used the subsample strategy (i.e. the presence points are repeatedly split into random training and testing subsets) as a form of replication. However, for genera with less than 30 locations (*Apodemus*, *Cannomys*, *Nesokia*) we instead used bootstrap (i.e. the training data is selected by sampling with replacement from the presence points, with the number of samples equalling the total number of presence points) as a form of replication. Although better suited for models with few occurrences, the fact that MaxEnt is allowed to test the model with occurrences that might have been used to train the model can slightly inflate AUC values. All models were run in software MaxEnt v3.3.3k [^7,9^](https://paperpile.com/c/87qg9w/ysx4T+J1wAJ) and evaluated using Receiver Operating Characteristic (ROC) curves.

**Model tuning and selection**

The careful tuning of the regularization multiplier (RM) when conducting Maxent modeling has also been reported to be essential to avoid model overfitting (Radosavljevic and Anderson, 2014) therefore we ran models with different RM values (1 and 2) to identify the best RM for the final predictive models. The RM values tested were based on possible overfitting for models with RM < 1, while models with RM = 2 can generally correspond to vegetation types where the species can occur and possible underfitting can occur for models with RM > 3 [^10^](https://paperpile.com/c/87qg9w/mBiZj) . Regularization multiplier values were tested for each spatially filtered data set and bias files, totalling twelve candidate models for each genus.

Model selection was based on, by order of importance: (1) minor omission rate (false negatives) using three thresholds (10P – 10 percentile training presence, MTP – Minimum training presence, ETSS – Equal training sensitivity and specificity) for binary predictions; (2) smaller AUCdiff (AUCtrain - AUCtest), as proxy for less overfitting [^11^](https://paperpile.com/c/87qg9w/zG4Eq); (3) higher predictive power (AUCtest). To understand if the best candidate models had similar predictions, we additionally calculated the Pearson correlation coefficient between the best RM1 and RM2 models.

**Environmental suitability for rodents across the Indian subcontinent**

We first used variable permutation importance to identify the most important environmental factors explaining the occurrence records for each genus. To obtain the overall environmental suitability for each weight class, we overlapped the environmental suitability maps (raw output) for genera belonging to the same weight class. An overall environmental suitability map including all genera was also calculated.

**Results**

**Model selection**

The best filtering strategy and background manipulation (i.e., bias file) was genus-specific. However, five out of nine final best models were based on the remove duplicate (RD) strategy data set while the bias file was present only in three of the selected models (Supplementary Table SM2.4). Models with RM = 1 had better performance when trained with lower number of occurrences (*Apodemus*, *Cannomys*, *Nesokia*, *Niviventer*), whereas for genera with higher sample size (*Bandicota*, *Golunda*, *Mus*, *Rattus*, *Tatera*) models with RM = 2 produced the best models. The best RM2 models for *Cannomys* and *Nesokia* were trained on only seven samples (Supplementary Table SM2.4) and showed considerable lack of detail, predicting large areas across the subcontinent as suitable for both genera (data not shown). In addition, the RM1 and RM2 models for the genus *Cannomys* revealed moderate correlation (r = 0.6), whereas all best models for the remaining genera revealed high correlation (r > 0.8). Based on the current known species range for species belonging to the genus *Cannomys* and *Nesokia*, we considered the best RM2 model predictions unlikely and therefore excluded the RM2 models from the best model set despite that they showed lower omission rates (Supplementary Table SM2.4). Overall, selected models tended to show lower omission rates, though slightly higher than theoretically expected, compared to default model settings. All models revealed a reasonable discrimination ability (AUCtest > 0.73).

**Environmental suitability for rodents across the Indian subcontinent**

Multiple environmental factors determined each genus’ ecological niche, but climatic factors were the most important for all genera except *Nesokia* (Supplementary Fig. SM2.5). Temperature variables were mainly important for six genera (*Apodemus*, *Cannomys*, *Bandicota*, *Golunda*, *Mus*, *Rattus*) and precipitation variables were considerably important for the genera *Niviventer*, *Rattus* and *Tatera*. Land cover information (including human settlements) had major influence on model predictions for all genera except for *Rattus*, *Mus* and *Bandicota*. Human population density had considerable importance to explain the occurrence of the genera *Apodemus*, *Bandicota* and *Golunda*. Lastly, topographic features (elevation) had higher contribution for the genera *Nesokia* and *Bandicota*.

Spatially, smaller body weight rodent occurrence (SRO) have higher probability of occurrence in the Central / Western Himalayas, south India and Sri Lanka. Similarly, south India and Sri Lanka were also more suitable for medium body weight rodents (MRO), but areas with high relative occurrence rate for these were also found in Western Pakistan, Central / Eastern Himalayas and Northeast India. Larger rodent occurrence (LRO) seem to have higher occurrence probability around the lower plains of the Indus, Ganga and Brahmaputra rivers, as well as along the Indian Eastern coast (Supplementary Fig. SM2.4 - 2.5). All maps produced were deposited in Dryad.

**Conclusion**

Our findings suggest that rodent genera preyed by small carnivores in the Indian subcontinent are likely to be influenced by many intricate factors, leading to a complex response to current environmental changes. While climatic variables were key factors explaining current rodent occurrence, land cover information had considerable influence on model predictions for all genera except for the genera *Rattus*, *Mus* and *Bandicota*. This could indicate that predictions for future rodent occurrence based only on climatic factors can be misleading and future land cover scenarios should be taken into account. In addition, human population density had considerable importance to explain the *Apodemus*, *Bandicota* and *Golunda* occurrence, suggesting that predicted increasing human population for the Indian subcontinent might also play a role on future rodent distribution.

Currently, prey distribution for small and medium sized carnivorous is likely to be heterogeneous across the subcontinent and predators most likely face different prey richness and different body weight prey in different biogeographic regions. For instance, desert areas and central India appeared, in general, to be less suitable for prey compared to areas such as the Himalayas or the Indian Eastern coast. It is worth noticing that some suitable areas (e.g. Himalayas) for small carnivore’s prey are already experiencing climatic modifications [^12^](https://paperpile.com/c/87qg9w/K6raN) and current distribution patterns might be disrupted in the future.

Caution is needed in the interpretation of our results because the models were built pooling occurrence records from different species belonging to the same genus. It is likely that there is variability in the most important environmental factors for each species and therefore our results might be biased toward species with more occurrence records within each genus. Moreover, for some genera (i.e. *Apodemus*, *Cannomys*, *Nesokia*) our models were built based on a limited sample size, although MaxEnt models have been able to extract useful information from a low number of occurrence records [^13^](https://paperpile.com/c/87qg9w/t9DWQ). During this study we verified that information on species distributions and population dynamics (crucial baseline information for prey and predator conservation actions) is rare across the Indian Subcontinent. We therefore hope that our results can be integrated in future large scale studies and guide future research efforts.

**Figure SM2.1** - Species adult body mass per genus. Lines represent division between weight classes.
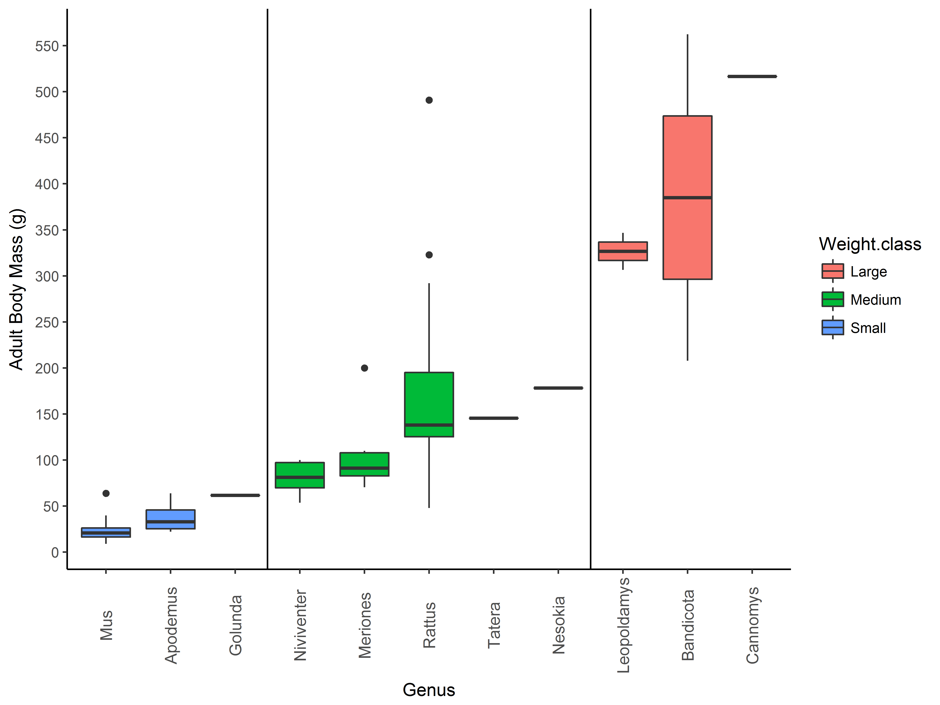


**Figure SM2.2** - Occurrence records used to model rodent occurrence across the Indian subcontinent after removing duplicate records( RD) within cells (~10km) or randomly removing locations from over-sampled administrative areas to match average point density between sampled areas (RDbal).
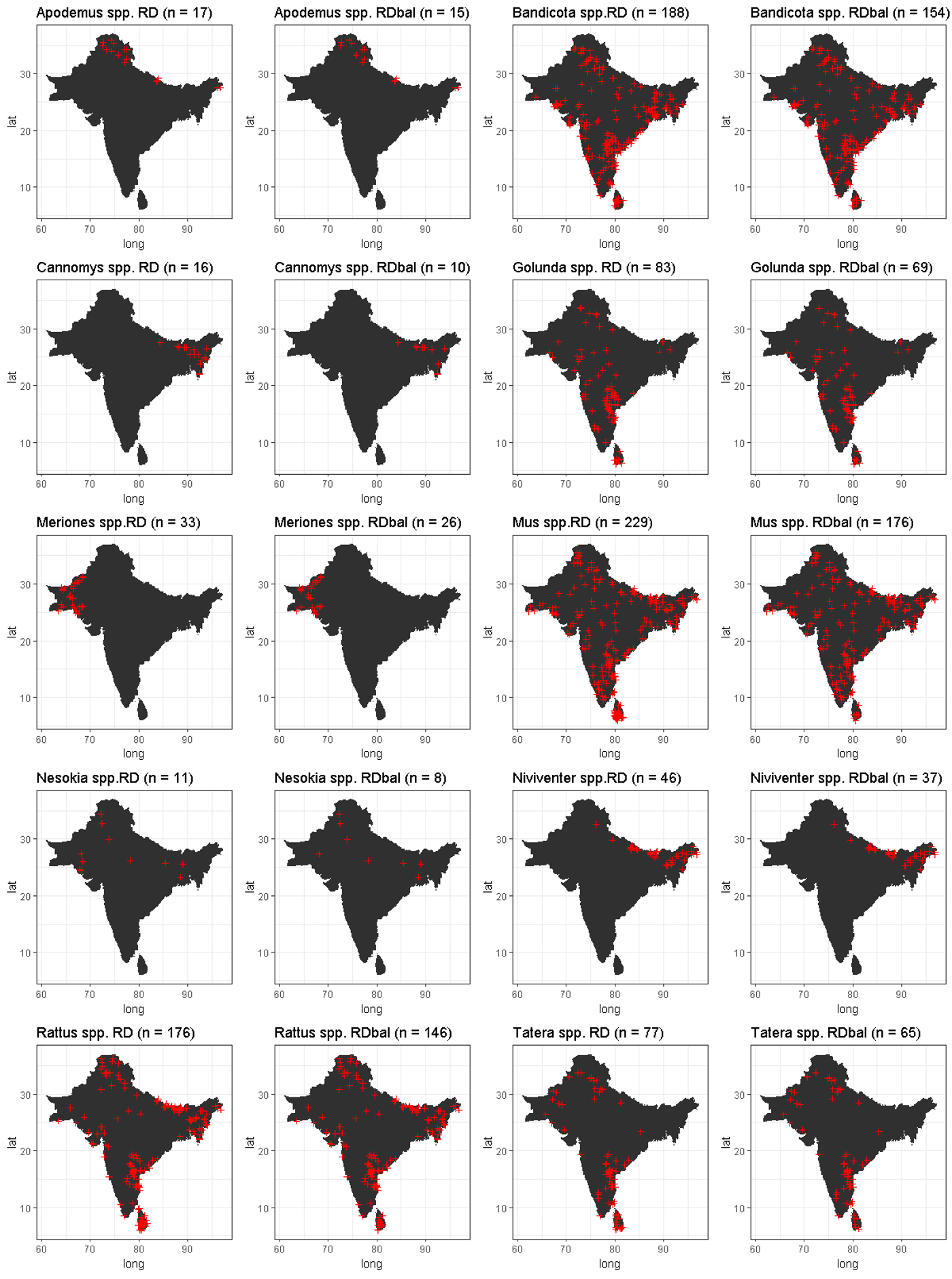


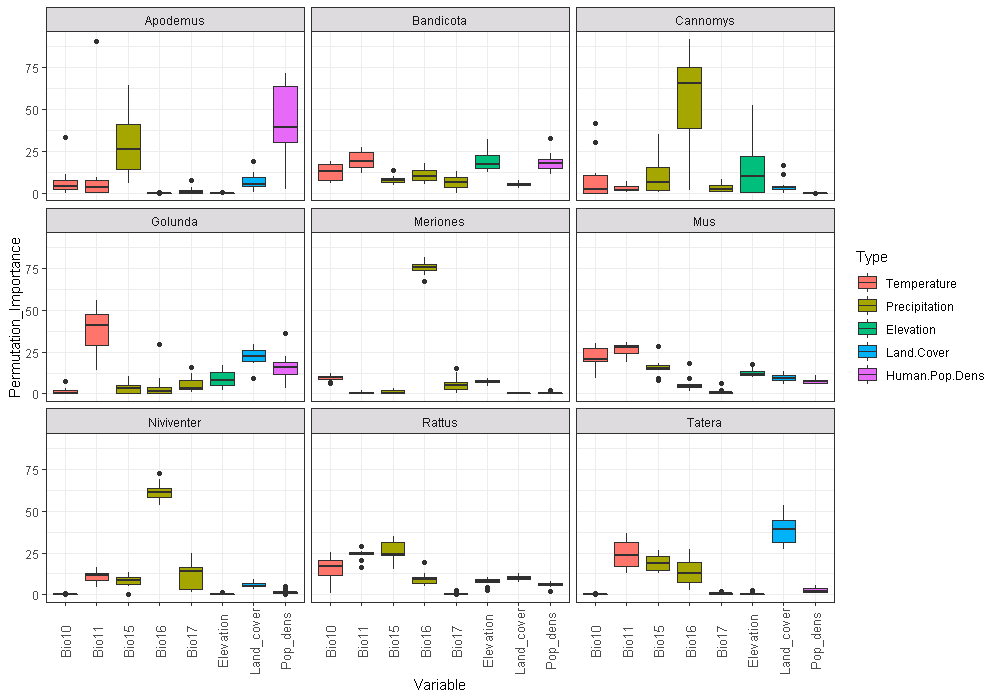


**Figure SM2.3** – Variable permutation importance for each genus. The values presented are for the best model (10 runs for each model). Variable importance is calculated by randomly permuting the values of that variable among the training points (both presence and background) and measuring the resulting decrease in training AUC. Values are normalized to give percentages. Higher percentages indicate that the model depends considerably on that variable.


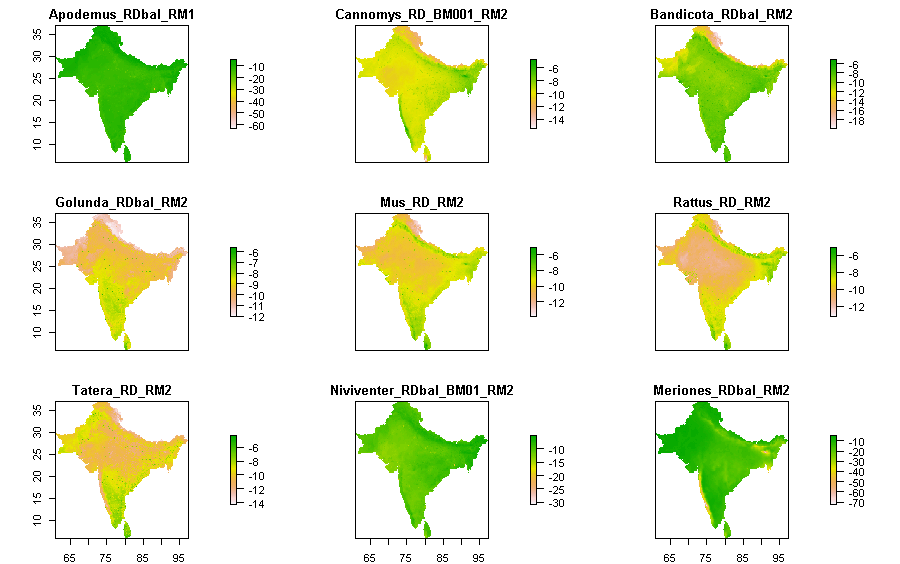


**Figure SM2.4** – Log-transformed Maxent raw output (log(ROR)) showing occurrence rate for each genus. Predictions are an average for 10 model replicates.


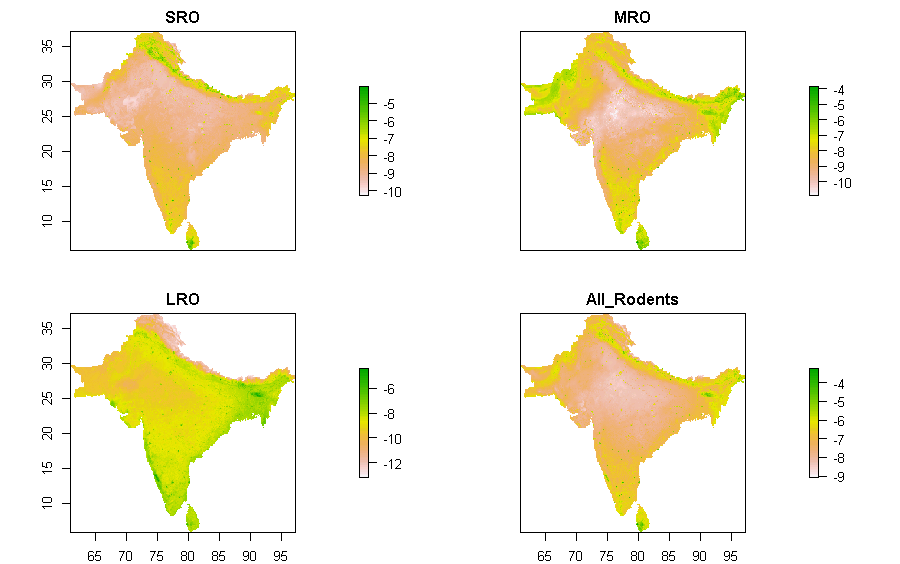


**Figure SM2.5** – Log-transformed Maxent raw output (log(ROR)) per body weight group. Occurrence for each group is calculated by summing raw output from the best model predictions for genera belonging to each body weight group.

**Data sources in table SM2.1**

1. Adhya, T., 2015. Habitat use and diet of two sympatric felids - the Fishing cat (Prionailurus viverrinus) and the Jungle cat (Felis chaus) - in a human-dominated landscape in suburban Kolkata. National Centre for Biological Sciences Tata Institute of Fundamental Research.
2. Aroon, S., 2008. Diet and habitat use of viverrid group at Sakaerat Environmental Research Station, Nakhon Ratchasima. Suranaree University of Technology.
3. Chuang, S.A., Lee, L.L., 1997. Food habits of three carnivore species (Viverricula indica, Herpestes uwa, and Melogale moschata) in Fushan Forest, northern Taiwan. J. Zool. London 243, 71–79. doi:10.1111/j.1469-7998.1997.tb05757.x
4. Grassman Jr, L.I., Tewes, M.E., Silvy, N.J., Kreetiyutanont, K., 2005. Spatial organization and diet of the leopard cat (Prionailurus bengalensis) in north-central Thailand. J. Zool. 266, 45–54.
5. IUCN, 2015. International Union for Conservation of Nature [WWW Document]. URL http://www.iucnredlist.org/
6. Jaeger, M.M., Haque, E., Sultana, P., Bruggers, R.L., 2007. Daytime cover, diet and space-use of golden jackals (Canis aureus) in agro-ecosystems of Bangladesh. Mammalia 71, 1–10.
7. Mukherjee, S., Goyal, S.P., Johnsingh, A.J.T., Pitman, M.R.P.L., 2004. The importance of rodents in the diet of jungle cat (Felis chaus), caracal (Caracal caracal) and golden jackal (Canis aureus) in Sariska Tiger Reserve, Rajasthan, India. J. Zool. 262, 405–411.
8. Rabinowitz, A., 1990. Notes on the Behavior and Movements of Leopard Cats, Felis bengalensis, in a Dry Tropical Forest Mosaic in Thailand. Biotropica 22, 397–403. doi:10.2307/2388557
9. Rajaratnam, R., Sunquist, M., Rajaratnam, L., Ambu, L., 2007. Diet and habitat selection of the leopard cat (Prionailurus bengalensis borneoensis) in an agricultural landscape in Sabah, Malaysian Borneo. J. Trop. Ecol. 23, 209–217.
10. Rana, S.A., Smith, S.M., Javed, M., Siddiqui, I., 2005. Scat Analysis of Small Indian Mongoose (Herpestes auropunctatus) Feeding on Fauna of Some High and Relatively Low Input Crop Fields. Int. J. Agric. Biol. 7, 7–5.
11. Shehzad, W., Riaz, T., Nawaz, M.A., Miquel, C., Poillot, C., Shah, S.A., Pompanon, F., Coissac, E., Taberlet, P., 2012. Carnivore diet analysis based on next-generation sequencing: Application to the leopard cat (Prionailurus bengalensis) in Pakistan. Mol. Ecol. 21, 1951–1965.
12. Su, S.U., Sale, J., 2007. Niche differentiation between Common Palm Civet Paradoxurus hermaphroditus and Small Indian Civet Viverricula indica in regenerating degraded forest , Myanmar. Small Carniv. Conserv. 36, 30–34.
13. Vanak, A., Gompper, M., 2009. Dietary niche separation between sympatric free-ranging domestic dogs and Indian foxes in central India. J. Mammal. 90, 1058–1065.
14. Xiong, M., Wang, D., Bu, H., Shao, X., Zhang, D., Li, S., Wang, R., Yao, M., 2017. Molecular dietary analysis of two sympatric felids in the Mountains of Southwest China biodiversity hotspot and conservation implications. Sci. Rep. 7, 41909. doi:10.1038/srep41909

**References**

1. [Molur, S. *et al.* *Status of South Asian Non-volant Small Mammals*. (2005).](http://paperpile.com/b/87qg9w/GmW9v)

2. [Jones, K. E. *et al.* PanTHERIA: a species-level database of life history, ecology, and geography of extant and recently extinct mammals. *Ecology* vol. 90 2648–2648 (2009).](http://paperpile.com/b/87qg9w/zRfaD)

3. [Arregoitia, L. D. V., Verde Arregoitia, L. D., Blomberg, S. P. & Fisher, D. O. Phylogenetic correlates of extinction risk in mammals: species in older lineages are not at greater risk. *Proceedings of the Royal Society B: Biological Sciences* vol. 280 20131092–20131092 (2013).](http://paperpile.com/b/87qg9w/HesB9)

4. [Anderson, R. P. & Gonzalez, I. Species-specific tuning increases robustness to sampling bias in models of species distributions: An implementation with Maxent. *Ecological Modelling* vol. 222 2796–2811 (2011).](http://paperpile.com/b/87qg9w/hbHLw)

5. [Phillips, S. J. *et al.* Sample selection bias and presence-only distribution models: implications for background and pseudo-absence data. *Ecological Applications* vol. 19 181–197 (2009).](http://paperpile.com/b/87qg9w/K6FJW)

6. [Kramer-Schadt, S. *et al.* The importance of correcting for sampling bias in MaxEnt species distribution models. *Diversity and Distributions* vol. 19 1366–1379 (2013).](http://paperpile.com/b/87qg9w/fwpra)

7. [Phillips, S. J., Anderson, R. P. & Schapire, R. E. Maximum entropy modeling of species geographic distributions. *Ecological Modelling* vol. 190 231–259 (2006).](http://paperpile.com/b/87qg9w/J1wAJ)

8. [Elith, J. *et al.* Novel methods improve prediction of species’ distributions from occurrence data. *Ecography* vol. 29 129–151 (2006).](http://paperpile.com/b/87qg9w/OtrCa)

9. [Phillips, S. J. & Dudík, M. Modeling of species distributions with Maxent: new extensions and a comprehensive evaluation. *Ecography* vol. 0 080328142746259–??? (2008).](http://paperpile.com/b/87qg9w/ysx4T)

10. [Radosavljevic, A. & Anderson, R. P. Making better Maxentmodels of species distributions: complexity, overfitting and evaluation. *Journal of Biogeography* vol. 41 629–643 (2014).](http://paperpile.com/b/87qg9w/mBiZj)

11. [Warren, D. L. & Seifert, S. N. Ecological niche modeling in Maxent: the importance of model complexity and the performance of model selection criteria. *Ecol. Appl.* **21**, 335–342 (2011).](http://paperpile.com/b/87qg9w/zG4Eq)

12. [Shrestha, U. B., Gautam, S. & Bawa, K. S. Widespread climate change in the Himalayas and associated changes in local ecosystems. *PLoS One* **7**, e36741 (2012).](http://paperpile.com/b/87qg9w/K6raN)

13. [Pearson, R. G., Raxworthy, C. J., Nakamura, M. & Townsend Peterson, A. ORIGINAL ARTICLE: Predicting species distributions from small numbers of occurrence records: a test case using cryptic geckos in Madagascar. *Journal of Biogeography* vol. 34 102–117 (2006).](http://paperpile.com/b/87qg9w/t9DWQ)
